# Optimization of Wild Blackthorn (*Prunus spinosa* L.) Purée Drying: Effect of Egg Albumen Concentration Using Response Surface Methodology

**DOI:** 10.3390/foods15142449

**Published:** 2026-07-10

**Authors:** Silviu Măntăilă, Nicoleta Balan, Mihaela Cotârleț, Oana Viorela Nistor, Doina Georgeta Andronoiu, Dănuț-Gabriel Mocanu

**Affiliations:** Faculty of Food Science and Engineering, “Dunarea de Jos” University of Galati, 800008 Galati, Romania; sm193@student.ugal.ro (S.M.); nicoleta.balan@ugal.ro (N.B.); mihaela.cotarlet@ugal.ro (M.C.); oana.nistor@ugal.ro (O.V.N.); georgeta.andronoiu@ugal.ro (D.G.A.)

**Keywords:** polyphenols, antioxidant activity, drying, wild blackthorn, extraction, color

## Abstract

*Prunus spinosa* L. (wild blackthorn) purée is an effective, nutrient-dense matrix that is rich in polyphenols, dietary fiber, and ascorbic acid, possessing significant potential as a functional food ingredient. This study investigated the technological valorization of wild blackthorn purée using convective and infrared-assisted foam-mat drying, evaluating the impact of temperatures (50, 60, and 70 °C) on bioactive profiles, antioxidant activity, color parameters, and powder flowability. Results indicated that the Midilli and Page models proved the best fit for the experimental drying kinetics, with higher effective moisture diffusivity (D_eff_) observed in infrared-assisted systems. Convective foam-mat drying (60 °C, 5% egg albumen) resulted in a higher anthocyanin content compared to the control (0% egg albumen), corresponding to a relative increase of 20.8% based on HPLC analysis. Under infrared drying at 60 °C, the 10% egg albumen formulation showed a relative increase of 7.8% compared to the corresponding control. Samples without egg albumen (0% EA) exhibited baseline polyphenolic content under both drying techniques. Samples obtained without egg albumen (0% EA) under optimized conditions exhibited improved color stability and maintained polyphenolic content. Overall, the results highlight the combined effect of drying method, temperature, and egg albumen concentration on the quality of blackthorn purée powders under the studied conditions.

## 1. Introduction

Wild blackthorn, belonging to the *Prunus* clade of the *Rosaceae* family, is one of the most cultivated thorny shrubs, particularly in the form of hybrids [1]. The blue-black fruits are rich in polyphenolic compounds, including flavanols, phenolic acids, and anthocyanins [2,3], along with a high content of vitamin C [4], fiber, and polysaccharides [5].

In recent years, modern consumers have shown a growing interest in formulations in which synthetic colorants and flavoring agents are replaced with natural alternatives derived from plant sources [6]. In addition, inexhaustible sources of bioactive compounds with antioxidant, anti-inflammatory, antidiabetic, and antibacterial activity, such as wild blackthorn, have been reported in numerous studies [2,4,5,7]. Nistor et al. (2023) [2] highlighted the potential of wild blackthorn skin extract in inhibiting the activity of enzymes associated with postprandial diabetes (α-amylase and α-glucosidase) and tyrosinase activity, attributed to its polyphenolic profile, which is rich in vanillic acid, gallic acid, chlorogenic acid, myricetin, catechin, and the glycosidic forms of peonidin and cyanidin.

Due to the fruit’s seasonal availability and high water content, drying the wild blackthorn purée enhances the stability and usability of the resulting powder and facilitates the development of high-value products that may serve as coloring, flavoring, and preserving agents, owing to their complex chemical composition [4,7]. Therefore, to ensure the practical applicability of this powder as a colorant, it is necessary to select a drying technique that preserves pigments, such as anthocyanins, which are known for their low thermal stability [8].

Several drying techniques have been reported in the literature for blackthorn fruit, such as freeze-drying [2,9], hot air convection drying [9], or foam-mat drying using different methods: microwave, hot air convection, and natural drying [4].

Foam-mat drying is an effective technique for preserving thermally sensitive bioactive compounds (such as anthocyanins) by creating a porous foam structure that enhances heat and mass transfer, thereby reducing drying time [10,11]. The foam matrix also helps protect bioactive compounds from heat and oxidation, while the resulting powders exhibit good rehydration properties and extended shelf life [12]. Compared with conventional drying methods, foam-mat drying is a cost-effective approach that operates at lower temperatures and has been successfully applied to a wide range of fruit and vegetable products [4,12]. The efficiency of foam-mat drying is strongly influenced by foam characteristics, including foam stability, the type and concentration of the foaming agent, the selected drying conditions, and possible interactions between the puree constituents and the foaming agent, which may promote reactions such as the Maillard reaction.

However, limited studies have addressed the optimization of foam-drying conditions for wild blackthorn fruits and their combined effect on color attributes and polyphenolic stability.

Infrared drying was selected due to its rapid heating mechanism and potential to enhance moisture removal while minimizing processing time, making it a relevant comparison to conventional convective and foam-mat systems [13,14].

The present study followed two main objectives: optimizing the drying conditions of wild blackthorn purée using Response Surface Methodology (RSM) by applying a Three-Level Factorial Design on the CIELAB parameters of the dried powders and performing a comparative analysis of polyphenolic compounds (total phenolic content (TPC), total anthocyanin content (TAC), and total flavonoid content (TFC)) and antioxidant activity of the powders to evaluate the efficiency of foam-mat drying.

The drying techniques applied were hot air convection (CD) and infrared (IR) drying. For each method, 13 experimental runs were conducted using two independent variables: temperature (50, 60, and 70 °C) and egg albumin concentration (0, 5, and 10%). Subsequently, based on the optimization and validation of the drying conditions, the PCA results, and polyphenolic content, the powders were subjected to polyphenolic profile characterization by high-performance liquid chromatography (HPLC).

The novelty of this study consists of an integrated approach combining response surface optimization, PCA-based multivariate analysis, and HPLC characterization applied to foam-mat and infrared drying of wild blackthorn purée.

## 2. Materials and Methods

### 2.1. Raw Material

Wild blackthorn (*Prunus spinosa* L.) fruits were harvested at the end of November 2024 from the Camnița forest Constantinești village, Brăila, Romania. The fruits were stored at −20 °C until use. Fresh eggs were purchased from a local store in Galați, Romania.

### 2.2. Reagents

The reagents used for the global phytochemical characterization and antioxidant activity, as well as the phenolic compound standards used in HPLC analysis, were the same as those described in our previous study [15].

### 2.3. Purée Preparation

A 1 kg batch of frozen fruits (−20 °C) was used for each sample. The fruits were thawed under refrigeration conditions at 4 °C for 24 h and manually separated from the seeds. The pulp, together with the skin, was blended into a purée using a vertical blender (TEFAL Quickchef+ HB67G830, 0.8 L, 1000W, Bucharest, Romania) for 5 min at constant speed. For the foam-mat drying process, the fresh egg white albumen was whipped using the same device equipped with a balloon whisk attachment at maximum speed (Turbo mode) for 5 min. Following egg white whipping (density: 1.04 ± 0.02 g/cm^3^), foam density, overrun, foam stability, and drainage were determined according to the methods described by Hossain et al. (2024) [12] and Bayram (2024) [4]. The resulting foam exhibited a foam density of 0.240 ± 0.01 g/cm^3^, an overrun of 333.33 ± 1.25%, a foam stability of 84.22 ± 0.77%, and a drainage ratio after 1 h of 0.45 ± 0.05.

The obtained foam was added to the purée at concentrations of 5% and 10% (*w*/*w*, based on purée mass). The mixture was then gently folded for 1–2 min using 20 uniform folding strokes to preserve the air bubbles.

### 2.4. Drying Techniques

**Figure 1:**
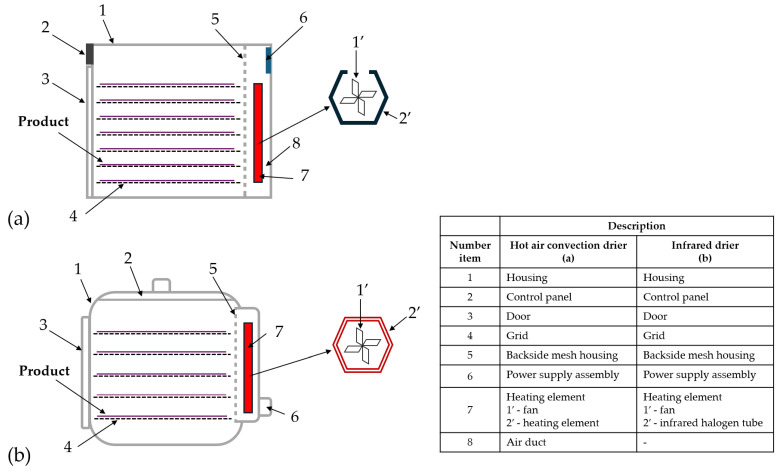
Schematic representation of the component elements of both drying equipment used.

Approximately 500 g ± 5 g of *Prunus spinosa* purée containing 0%, 5% and 10% egg albumin (EA) was spread evenly on a baking paper, forming a thin layer of 0.50 ± 0.05 cm, and processed using two different drying techniques—hot air convection drying (CD) performed using a Hendi Profi Line food dehydrator, 1000 W (Brașov, Romania) and infrared drying (IR) achieved with a Concept SO4000 Infra 500 W equipment (Choceň, Czech Republic)—at temperatures of 50 °C, 60 °C, and 70 °C (Figure 1).

For the IR dryer, the drying chamber is fitted with an infrared halogen tube and a conventional metal coil heating element (Figure 1b). The wavelength ranges from 0.8 μm to 3.0 μm. The equipment consumes a maximum total power of 500 W. The Concept SO4000 has 5 perforated mesh trays measuring 30 cm × 24 cm. Because the halogen tube is back-mounted, the horizontal distance between the emitting surface and the purée samples ranges from 20 mm at the back of the trays to roughly 260 mm at the front.

For both drying systems, the air velocity was maintained at 1.2 m·s^−1^, and the relative humidity was approximately 13% under the experimental conditions.

### 2.5. Mathematical Modeling of Blackthorn Purée Drying

For mathematical modeling, 30.00 ± 0.15 g of samples was spread in a 0.50 ± 0.01 cm on baking paper and weighed using a digital balance (Precisa EP–125SM, Iași, Romania) every 30 min. Equilibrium moisture content (M_e_) was determined from the final constant mass condition. The dried samples were ground using an electric grinder (Coffee grinder Heinner HCG-150SS; Heinner, Network One Distribution, Bucharest, Romania). Each batch of approximately 20–30 g was ground for approximately 20–30 s until a fine and homogeneous powder was obtained. The powders were stored in hermetically sealed glass jars at room temperature (20 ± 1 °C) in the dark until further analysis.

For the graphical representation of the drying curves, the moisture ratio (MR) and drying rate (DR) were calculated according to Equations (1) and (2), as described by Stan (Boldea) et al. (2025) [16]. Moisture content was expressed on a dry basis (kg water per kg dry matter).

(1) $$MR\;\left(dimensionless\right)=\frac{M_{t}-M_{e}}{M_{0}-M_{e}}$$

where $M_{t}$ is the moisture content of the sample at time t (kg·kg^−1^), $M_{e}$ is the equilibrium moisture content reached (kg·kg^−1^), and $M_{0}$ is the initial moisture content of the sample (kg·kg^−1^).

(2) $$DR=\frac{M_{t}-M_{t+∆t}\;}{∆t}$$

where $M_{t+∆t}$ is the moisture content at time t + ∆t, and ∆t = 30 min and represents the time interval between two consecutive measurements.

To identify the best-fit mathematical model for drying of blackthorn purée with different EA concentrations, nonlinear regression equations corresponding to the following drying models were evaluated, selected based on their wide application in the literature for foam-mat drying kinetics of various food matrices and their proven ability to accurately describe the drying behavior of fruit and plant-based materials: Lewis with Equation (3), Page with Equation (4), Henderson & Pabis with Equation (5), Logarithmic with Equation (6), Wang & Singh with Equation (7), Midilli with Equation (8) and Diffusion Approach with Equation (9) [14,15,17].

(3) $$MR=\exp\left(-kt\right)$$

(4) $$MR=exp(-kt^{n})$$

(5) $$MR=a\exp\left(-kt\right)$$

(6) $$MR=\mathrm{a exp}\left(-kt\right)+c$$

(7) $$MR=1+at+bt^{2}$$

(8) $$MR=a\exp\left(-kt^{n}\right)+bt$$

(9) $$MR=a\exp\left(-kt\right)+\left(1-a\right)exp(-kbt)$$

For each model, specific coefficients (k, n, a, b, c) were determined, and statistical parameters—root mean square error (RMSE), reduced chi-square (χ^2^), and coefficient of determination (R^2^)—were calculated and used as the main indicators for identifying the best-fitting mathematical model for both drying techniques (CD and IR) [15].

### 2.6. Effective Moisture Diffusivity (D_eff_) and Activation Energy (E_a_)

Equation (10), according to Fick’s second law of diffusion, was used to calculate the effective moisture diffusivity (D_eff_) during the drying process of blackthorn purée at different levels of EA concentrations [16].

(10) $$MR=\frac{8}{\pi^{2}}\sum_{n=0}^{\infty }\frac{1}{{\left(2n1\right)}^{2}}exp\left[{-(2n+1)}^{2}\times \frac{\pi^{2}\left(D_{eff}\times t\right)}{{4L}^{2}}\right]$$

where MR is the moisture ratio of the analyzed sample, t is the time (s), L is the half-thickness of the material being dried (m), corresponding to symmetric two-sided slab geometry, and D_eff_ is the effective moisture diffusivity (m^2^·s^−1^).

By converting Equation (10) into ln (MR), it was possible to calculate the value of D_eff_ by plotting ln(MR) versus drying time. According to the Arrhenius equation (Equation (11)), based on the relationship established between the calculated D_eff_ and the three temperatures studied, the activation energy (E_a_) was calculated [15].

(11) $$D_{eff}=D_{0}exp\left(-\frac{E_{a}}{R\times T}\right)$$

where D_0_ is the pre-exponential factor of the Arrhenius equation (m^2^·s^−1^), E_a_ is the activation energy (kJ·mol^−1^), T is the absolute temperature (K), and R is the universal gas constant (8.31451 J·mol^−1^·K^−1^).

### 2.7. Extraction and Phytochemical Characterization of Blackthorn Powders

The hydroalcoholic extracts were obtained following the method described by Stan (Boldea) et al. (2025) [16]. Dried powder (1.00 g ± 0.01 g) was weighed (Precisa EP–125SM digital balance, Iasi, Romania) and mixed with 9 mL of ethanol: water solution (70:30, *v*/*v*). Extraction of bioactive compounds was performed in an ultrasonic bath (Digital Ultrasonic Bath Mod. DU-32; 131 ARGOLAB; Capri, Italy) at 35 ± 1 °C for 30 min (40 kHz, 100 W). The supernatant obtained after centrifugation (Universal 320R Centrifuge, Hettich; Germany) at 7000 rpm for 10 min at 4 °C [18] was collected and used for assessing the total anthocyanin content (TAC), total polyphenolic content (TPC), total flavonoid content (TFC), antioxidant activity (DPPH), and chromatographic analysis (HPLC).

### 2.8. Phytochemical Characterization

#### 2.8.1. The Global Phytochemical Characterization

The global phytochemical characterization was achieved by spectrophotometric methods using a Biochrom Libra 22 UV/Visible spectrophotometer (Biochrom Ltd., Holliston, MA, USA), following the methods described by Balan et al. (2025) [19]. The supernatant obtained according to Section 2.7 was diluted 1:2 before analysis. TAC was expressed as mg cyanidin-3-O-glucoside per 100 g dry weight (mg C3G·100g^−1^ DW). TPC was expressed as gallic acid equivalents (GAE·g^−1^ DW), and TFC as quercetin equivalents (QE·g^−1^ DW).

#### 2.8.2. Antioxidant Activity

The antioxidant activity of the extracts was quantified based on their ability to scavenge the 2,2-diphenyl-1-picrylhydrazyl radical, according to the procedure applied by Tănase (Butnariu) et al. (2024) [20] with minor modifications. A freshly prepared DPPH solution (0.04 mg·mL^−1^) in methanol (HPLC grade) was used. Briefly, 0.10 mL of diluted sample (1:2, *v·v*^−1^) was mixed with 3.90 mL DPPH solution and incubated in the dark at 25 °C for 90 min. The absorbance was measured at 515 nm against a methanol blank. Antioxidant activity was expressed as µmol Trolox·g^−1^ DW using the calibration curve (y = 0.45x + 0.0075, R^2^ = 0.998).

### 2.9. Advanced Analysis of Extracts Using High-Performance Liquid Chromatography (HPLC)

The chromatographic analysis was performed according to the method described in our previous study [15]. The analysis was carried out using an Agilent 1200 system (Agilent Technologies, Santa Clara, CA, USA) system equipped with an autosampler (10 µL) with a flow rate of 1 mL·min^−1^, degasser, quaternary pump system, multi-wavelength detector, and column (250 × 4.6 mm, 5 µm particle size, Phenomenex, Torrance, CA, USA) with thermostat set up at 30 °C. The mobile phases consisted of 100% methanol (A) and 10% formic acid (B), and the following gradient elution was applied: 0–20 min: 9% A and 91% B, 20–30 min: 35% A and 65% B, 30–40 min: 50% A and 50% B, 40–45 min: 9% A and 91% B. Data analysis was performed automatically using Agilent ChemStation software, version Rev. B.04.03, based on the existing calibration curves for each polyphenolic compound. The results were expressed as mg·100 g^−1^ DW.

### 2.10. Powder Flow Properties

The bulk density (BD) and tap density (TD) were determined according to the method described by Ozdikicierler et al. (2014) [21]. Five grams of each sample was placed in a 25 mL graduated cylinder. The initial volume occupied by the sample was recorded as the bulk density (BD) expressed in g·mL^−1^ using Equation (12). To determine tapped density (TD), the cylinder was subjected to 300 mechanical taps (representing the moment of sample volume stabilization) at a constant tapping force (Equation (13)).

(12) $$Bulk\;density\;\left(BD\right),\;g\cdot {mL}^{-1}=\frac{M}{V_{0}}$$

(13) $$Tapped\;density\;\left(TD\right),\;g\cdot {mL}^{-1}=\frac{M}{V_{300}}$$

where M is the sample weight in g, V_0_ is the initial volume occupied by the sample in mL, and V_300_ is the final volume after 300 taps.

The obtained BD and TD values were used to calculate Carr’s compressibility index (CI) and the Hausner ratio (HR) using Equations (14) and (15), and the obtained results allowed the powders to be classified according to Gorle & Chopade (2020) [22].

(14) $$Carr\;Index\;\left(CI\right),\;\%=\frac{TD-BD}{TD}\times 100$$

(15) $$Hausner\;Ratio\;\left(HR\right),\;\left(dimensionless\right)=\frac{TD}{BD}$$

### 2.11. Color Parameters

**Figure 2:**
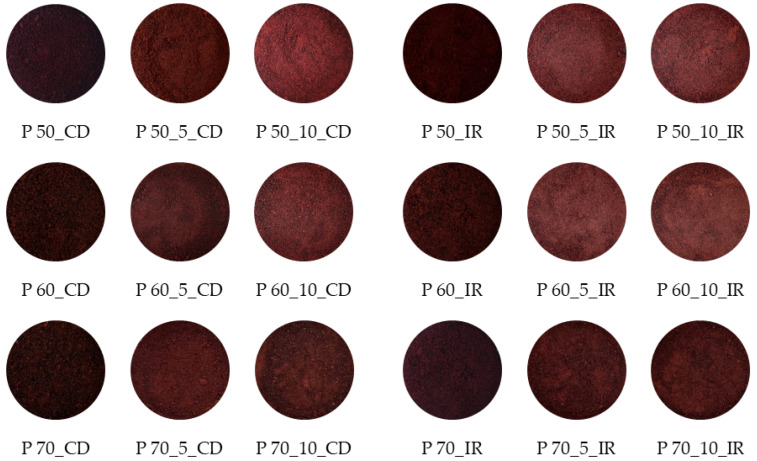
Powder obtained by CD and IR drying at different temperatures and EA concentrations.

Colorimetric measurements were performed on samples (fresh purée and the dried powders (Figure 2)) placed in a 60 mm glass Petri dish positioned on a white background. The material layer was kept at a consistent thickness of approximately 10 mm, and all samples were uniformly spread to ensure homogeneous surface condition analysis. Prior to measurements, the instrument was calibrated using the manufacturer’s standard white calibration plate. The 3nh NR110 precision colorimeter features an 8/d optical geometry and a 4 mm diameter measurement aperture. The colorimetric measurements were performed using a D-65 illuminant and a standard observer angle of 8°. The color coordinates: L* (lightness: 100 is white and 0 is black), a* (−60 to 0 is green intensity and 0 to 60 red intensity) and b* (−60 to 0 is blue intensity and 0 to 60 is yellow intensity) were determined using an NR110 precision colorimeter (Shenzhen 3nh Technology Co., Shenzhen, China).

From these primary color parameters, total color change (ΔE) and browning index (BI) were calculated, according to Equations (16) and (17) as reported by Ershadfarkar et al. (2023) [13].

(16) $$\Delta E={\left[{\left(L_{0}^{*}-L^{*}\right)}^{2}+{\left(a_{0}^{*}-a^{*}\right)}^{2}+{\left(b_{0}^{*}-b^{*}\right)}^{2}\right]}^{\frac{1}{2}}$$

(17) $$Browing\;Index\;\left(BI\right)=\frac{100\left[\left(\frac{a^{*}\;+\;1.75L^{*}}{5.645L^{*}\;+\;a^{*}\;-\;{3.012b}^{*}}\right)-0.31\right]}{0.17}$$

where $L_{0}^{*}$, $a_{0}^{*}$ and $b_{0}^{*}$ are the color parameters for the fresh purée samples; L*, a* and b* are the color parameters of powders dried under different conditions: L* (lightness/darkness), a* (redness: green to red), and b* (yellowness: blue to yellow).

### 2.12. Optimization of Drying Conditions Based on Blackthorn Powders’ Color Parameters

The optimization of color parameters L*, a*, b*, ΔE and BI as dependent variables, as a function of egg albumin concentration (EA) and temperature (t), was performed by applying Response Surface Methodology (RSM). A Three-Level Factorial Design was employed with two independent variables: egg white albumen concentration (EA, %) of 0, 5, and 10%, respectively; and drying temperature (t, °C) of 50, 60, and 70 °C. The experimental design generated by RSM (Table 4) consisted of a set of 13 experiments (runs) with 4 central points. The center-point values were suggested automatically by the experimental design software, which defined these replicates and required additional runs to ensure adequate model reliability. All experimental runs were randomized to minimize systematic error. The resulting design enabled the evaluation and optimization of color stability and appearance of the blackthorn powders under varying drying conditions. The experimental conditions were selected based on previous studies on foam-mat drying of various food matrices, which reported similar temperature ranges and formulation conditions as effective for drying performance [17,23,24].

### 2.13. Statistical Analysis

The determination of the specific coefficients (k, n, a, b, c) and statistical parameters (R^2^, RMSE, and χ^2^) for the seven mathematical drying models was performed using R-Console software version 4.4.0 (RCoreTeam, Vienna, Austria). The design of the experimental plans, optimization, and validation of the drying conditions were performed using Design-Expert software version 13.0 (Stat-Ease, Minneapolis, MN, USA). Significant differences between the results were established by performing analysis of variance (ANOVA), followed by Tukey’s test, at a 5% significance level (*p* < 0.05). Principal Component Analysis (PCA) and Pearson’s correlation were performed using Minitab software, version 19 (Romsym Data, Bucharest, Romania). All experiments, including drying experiments and all subsequent physicochemical and bioactive analyses, were conducted in triplicate (*n* = 3). Results are expressed as mean ± standard deviation (SD), with SD values calculated using Microsoft Excel.

## 3. Results and Discussion

### 3.1. Mathematical Modeling of Blackthorn Purée Drying

**Figure 3:**
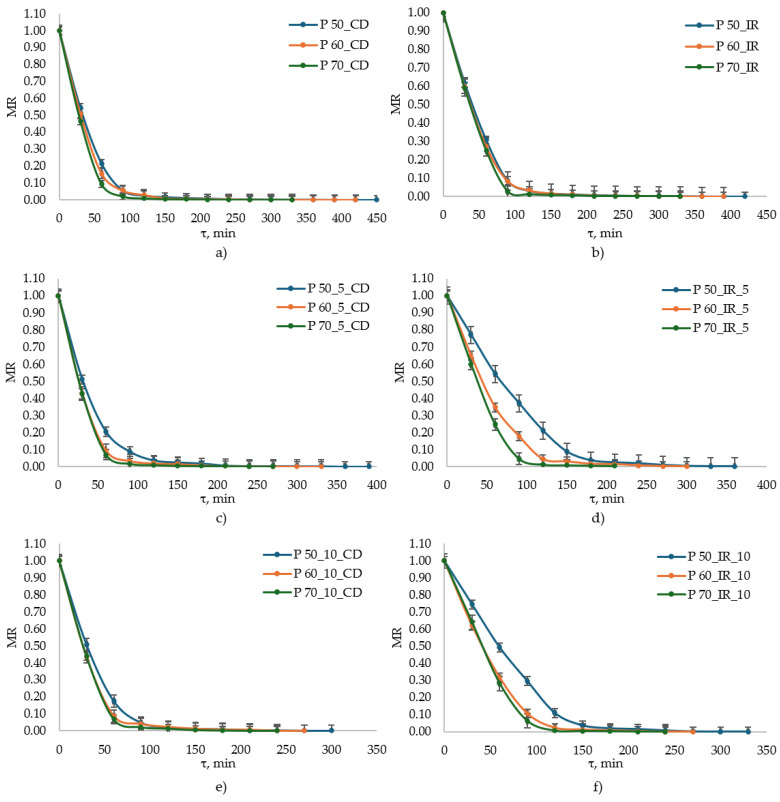
Moisture ratio (MR) variation depending on drying temperature and time for CD: (**a**) control sample, (**c**) sample with 5% EA, (**e**) sample with 10% EA and IR, (**b**) sample control, (**d**) sample with 5% EA, (**f**) sample with 10% EA.

The drying techniques applied in this study generated different moisture transfer rates within the food matrix, as can be seen in Figure 3. For both drying techniques, hot air convection (CD) and infrared (IR) drying, an increase in temperature led to a reduction in the total drying time required to reach constant mass. This behavior can be attributed to the enhanced diffusion mechanisms of moisture from the blackthorn purée to the drying environment, which promoted accelerated water evaporation.

Starting from an initial moisture content of the puree of 62.67 ± 0.03%, the two drying techniques applied at 50, 60, and 70 °C resulted in final powder moisture content ranging between 2.45 ± 0.26% and 4.87 ± 0.23%. The initial moisture content of the egg-albumin-enriched formulations at 5% and 10% EA was 71.38 ± 0.43% and 71.56 ± 0.88%, respectively. After drying, the moisture content of the powders ranged from 2.51 ± 0.26% to 3.52 ± 0.06% and from 1.85 ± 0.13% to 3.31 ± 0.26%, respectively. The end of the drying process was considered at constant mass (three consecutive identical values), corresponding to a final moisture content of approximately 2–5% under the investigated conditions.

As shown in Figure 3a,b, IR drying allowed a constant mass within 300–390 min, whereas the classical method (CD) required slightly longer times, between 300 and 420 min, to reach equilibrium moisture. The results obtained agree with the data reported by Puente-Díaz et al. (2020) [14], who reported similar trends during infrared-assisted and conventional convective drying of *Physalis* fruit purée at 60–90 °C.

Foam-mat drying is recognized as an efficient and economical technique that reduces overall drying time, with a high capacity to preserve biologically active compounds, resulting in a powder with sensory properties appreciated by consumers [25].

Analysis of Figure 3a–c indicates that foam-mat drying of *Prunus spinosa* purée led to reduced drying time by 14.29% at 70 °C and 20% at 50 °C for samples containing 5% EA, while for samples containing 10% EA, the drying time was reduced by 35.71% and 30%, respectively. This observation confirms the inverse relationship between temperature and drying time. Similar results were observed for IR, as shown in Figure 3d–f. Maciel et al. (2017) [17] investigated the foam-mat drying of guava pulp at 75, 80, and 85 °C using albumin concentrations of 4% and 8%. Like the results shown in Figure 3c,e, the authors reported a reduction in drying time with increasing temperature and albumin concentration, which was attributed to the incorporation of air that facilitated moisture diffusion and enhanced the mass transfer rate.

Diógenes et al. (2022) [23] reported a 64.29% reduction in drying time when increasing the temperature from 50 to 70 °C during the foam-mat drying of *Cumbeba* pulp, whereas in the present study, a 50% reduction was achieved with increasing temperature (Figure 3c). Ershadfarkar et al. (2023) [13] found that increasing infrared power produced faster moisture removal from black raspberries dried as a foam-mat layer, which facilitated a reduction in drying time due to the acceleration of the moisture transfer rate.

**Figure 4:**
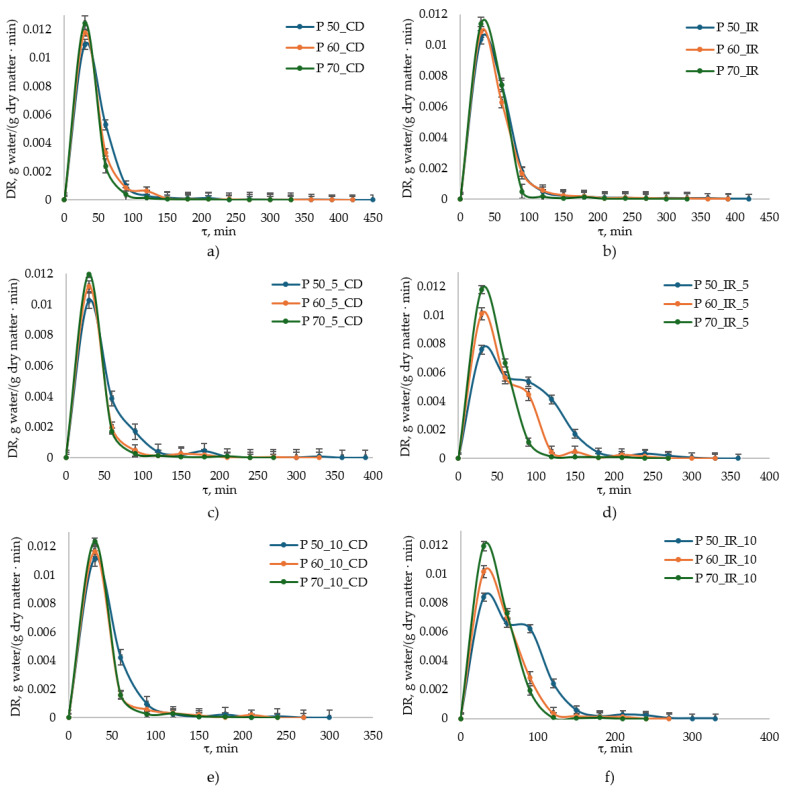
Drying ratio (DR) variation depending on drying temperature and time (τ) for CD: (**a**) control sample, (**c**) sample with 5% EA, (**e**) sample with 10% EA and IR, (**b**) sample control, (**d**) sample with 5% EA, (**f**) sample with 10% EA.

According to Figure 4, for both drying techniques applied and for both samples with and without EA addition, the first drying period was characterized by a high rate in the first 30 min due to the rapid evaporation of free water from the product surface, as observed at all temperatures applied [15]. The second drying period showed a lower drying rate caused by the reduced water diffusivity as the product surface dried, leading to the migration of the drying front towards the interior of the matrix and an increase in the internal resistance of water molecules. The same mechanism was reported by Diógenes et al. (2022) [23] during hot air foam-mat drying of *Cumbeba* pulp, by Ershadfarkar et al. (2023) [13] for infrared foam-mat drying of black raspberry, and by Puente-Díaz et al. (2020) [14] for both infrared-assisted and convective drying of Physalis fruit purée.

Experimental data on moisture content (MR) obtained during the drying of *Prunus spinosa* purée and its foam-mat variants with 5% and 10% EA using two drying techniques, CD and IR, were fitted to seven mathematical models to identify the best-fitting model for the experimental data.

Based on the nonlinear regression equations specific to each model, R^2^, RMSE, and χ^2^ were calculated to perform a comparative analysis and identify the most suitable model for foam-mat drying behavior of the samples. Table S1 presents the mathematical models evaluated, along with the corresponding statistical parameters and regression-specific coefficients obtained for each temperature under CD. The selection criteria for the best-fitting model were the highest R^2^ and the lowest RMSE and χ^2^ values. For CD (Table S1), R^2^ values ranged from 0.5612 to 0.9998, RMSE values from 0.1303 to 0.46 × 10^−2^, and χ^2^ values from 0.0212 to 0.21 × 10^−4^. According to Table S2, IR drying had an R^2^ ranging from 0.7333 to 0.9996, while RMSE and χ^2^ values varied between 0.0442–1.51 × 10^−2^ and 0.0245–2.49 × 10^−4^, respectively.

Residual analysis was indirectly assessed using RMSE and χ^2^ values, which quantify deviations between experimental and predicted moisture ratios. In addition, the agreement between experimental and predicted values (Figures S1 and S2) confirms the adequacy of the selected models.

Among the seven models evaluated, the three best-performing mathematical models were the Midilli, Page, and Logarithmic models (Figures S1 and S2). These models exhibited the highest R^2^ and the lowest RMSE and χ^2^ values and provided the best fit for describing the drying process of *Prunus spinosa* purée under all applied temperatures and drying techniques used, including foam-mat drying.

Maciel et al. (2017) [17] also reported that, for foam-mat drying (4% and 8% albumen) at 70–80 °C, the Midilli model, followed by the Page and Logarithmic models, best described the hot air convection drying of guava pulp, in agreement with the results obtained in Table S2. Diógenes et al. (2022) [23] reported similar results regarding the mathematical models identified as fitting the experimental results. Puente-Díaz et al. (2020) [14] observed that for infrared-assisted drying and non-assisted drying, respectively, the Midilli and Page models successfully fitted the experimental data, presenting the lowest values for RMSE (between 0.5 × 10^−2^–1.2 × 10^−2^, 0.2 × 10^−2^–2.1 × 10^−2^, respectively) and χ^2^ (between 0.1 × 10^−2^–0.4 × 10^−2^, and 0.1 × 10^−2^–0.2 × 10^−2^, respectively), while R^2^ ranged from 0.997 to 0.999 and 0.991 to 0.999.

A slight increasing trend in the Page model constant k was generally observed with increasing drying temperature and egg albumin concentration, indicating an enhancement of moisture transfer. For example, at 60 °C, k increased from 1.370 (0% EA) to 1.415 (5% EA) and 1.458 (10% EA). A similar tendency was observed for the Midilli model, particularly at higher temperatures (70 °C), as well as under certain infrared drying conditions (Tables S1 and S2).

However, this trend was not strictly monotonic across all experimental conditions; for instance, at 50 °C, k decreased from 1.427 (0% EA) to 1.404 (5% EA) and then increased to 1.478 (10% EA) (Tables S1 and S2). This non-uniform behavior can be attributed to complex interactions between matrix structure, foam stability, and heat and mass transfer mechanisms.

Figures S1 and S2 compare the experimental and predicted MR values for the three selected models (Midilli, Page, and Logarithmic). The distribution of points (experimental data) relative to the reference line indicates that the Midilli and Page models show a similar trend, confirming their suitability for describing the drying of *Prunus spinosa* purée, with different EA (0, 5, 10%) and different heat drying treatments (50, 60, 70 °C) under both CD and IR methods. In contrast, the Logarithmic model exhibited slightly larger deviation between the two MR models (predicted and experimental), indicating a weaker correlation. In the literature, the Midilli and Page models are consistently reported as the most appropriate for describing the foam-mat drying kinetics of fruit matrices [14,17,26].

### 3.2. D_eff_ and E_a_

Table 1 presents the D_eff_ and E_a_ values calculated from the experimental data obtained during the drying of *Prunus spinosa* purée and its foam-mat drying, applying two drying techniques (CD and IR). For convection drying, D_eff_ values varied between 0.76 and 1.23 × 10^−9^ m^2^·s^−1^ at 50, 60, and 70 °C. When 5% EA was added, an increase in the D_eff_ value was observed by 6.58% for 50 °C, 18.39% for 60 °C, and 0.81% for 70 °C. Increasing the foaming agent concentration to 10% EA further reduced the drying times, which was reflected in higher D_eff_ values, increasing by 34.57%, 15.53%, and 26.62%, respectively, at the applied temperatures. Rajkumar et al. (2007) [27] reported a 7.19% increase in D_eff_ values for convection drying of mango pulp at 60 °C when using 10% egg albumin and 0.5% methylcellulose as foaming agents.

Previous studies on foam-mat drying of various food matrices have demonstrated that higher drying temperatures and concentrations of foaming agent enhance effective moisture diffusivity (D_eff_). This process is explained by increasing molecular agitation of water and increased diffusion in the drying medium. The increase in temperature, together with air incorporation, increases mass transfer by improving contact between the drying agent and the surface of the material. Diógenes et al. (2022) [23] showed that, when applying different thicknesses (0.5–1.5 cm) during foam-mat drying, the D_eff_ value increases. The obtained results are in accordance with those reported in the literature for the dried layer with a thickness of 0.5 cm.

IR drying exhibited the same trend of increasing D_eff_ value with increasing temperature, and increasing EA content was observed. Moreover, the IR techniques generally resulted in higher effective moisture diffusivity compared to CD, as shown in Table 1, confirming the superior heat transfer efficiency of this drying technique.

The activation energy (E_a_) values decreased with increasing foaming agent concentration, from 22.30 to 16.83 kJ·mol^−1^ for CD drying. For IR drying, a similar decreasing pattern was observed, with slightly lower values than CD, ranging from 20.65 to 15.30 kJ·mol^−1^ (Table 1). A similar value was reported by Diógenes et al. (2022) [23] for E_a_ of 25.21 kJ·mol^−1^ following foam-mat drying of *Cumbeba* pulp, with a layer thickness of 0.5 cm, and the value of 15.12 kJ·mol^−1^ reported by Thuy et al. (2025) [11] for foam-mat drying of *Lacuma*.

### 3.3. Powder Characteristics

The use of EA as a foaming agent in hot air convection drying (CD) of *Prunus spinosa* purée resulted in significantly lower values of bulk and tapped densities compared with samples without EA, while the Carr Index (CI) and Hausner Ratio (HR) increased. In contrast, infrared drying (IR) led to a slight improvement in the two flowability indices, observed particularly in samples with 5% EA, compared to the CD method.

According to Table 2, foam-mat drying with 10% EA using the CD method led to an increase in bulk density and tapped density as temperature increased from 50 to 70 °C, ranging from 0.509 ± 0.009 to 0.566 ± 0.011 g/mL for BD and from 0.657 ± 0.008 to 0.757 ± 0.022 g/mL for TD. Similar trends were reported by Kaba et al. (2025) [24] during foam-mat drying (10% EA, 1% carboxymethylcellulose, and 10 maltodextrin) of *Cornelius* cherry at temperatures between 50 and 80 °C.

The same trend was observed for IR-dried powders (Table 2), with slightly lower values recorded at 50 and 60 °C compared to CD. The increase in flowability indices (CI and HR) with temperature can be explained by the reduction in moisture content, which increases cohesiveness due to interparticle forces and the interlocking mechanism [24].

Powders with acceptable flowability were obtained at 50 and 60 °C without a foaming agent due to the low CI and HR values, while powders dried at 70 °C showed poor flowability (Table 4). The use of 5% EA resulted in powders with poor flowability, while 10% EA produced a slight improvement, classifying these samples as “passable” according to the classification by Gorle & Chopade (2020) [22].

Under both drying methods, the high temperatures (70 °C) lead to high values for CI and HR, in accordance with previous studies. This fact is attributed to the denaturation of the protein structure of the foaming agent and the formation of irregular particles caused by rapid water evaporation from the material during drying. Therefore, the drying method significantly influenced the rheological properties of the powders. IR drying produced powders with slightly improved flowability compared with CD, likely due to rapid moisture removal, damage to the protein structure, and loss of air incorporated in the matrix during drying [13]. In addition, insufficient stability of the foaming agent leads to powders with reduced porosity and increased density, thereby causing an increase in CI and HR values [12].

### 3.4. Bioactive Compounds of the Powder

The extracts obtained from powders dried by CD and IR were analyzed for total anthocyanin content (TAC), total polyphenol content (TPC), total flavonoid content (TFC), and antioxidant activity (DPPH). All the assays were performed in triplicate (*n* = 3) for the purpose of comparative analysis of the efficiency of foam-mat drying of *Prunus spinosa* purée, particularly focusing on the protection of heat-sensitive compounds, such as anthocyanins.

As shown in Table 3, the TAC values obtained for the fresh sample (P Fresh) and the nine powders obtained after CD ranged from 11.57 ± 0.55 to 62.42 ± 2.58 mg C3G·100 g^−1^ DW. The highest values for this dependent variable (TAC) were obtained for the sample coded P 50_CD (26.32 ± 1.02 mg C3G·100 g^−1^ DW), followed by P 70_CD (18.80 ± 0.59 mg C3G·100 g^−1^ DW), both powders without EA addition. However, at the intermediate temperature of 60 °C, the sample with 5% EA led to the highest TAC value (16.36 ± 0.27 mg C3G·100 g^−1^ DW), compared with the sample without EA addition (P 60_CD) and the sample with 10% EA addition (P 60_10_CD), indicating the practical applicability of foam-mat drying and its effectiveness in preserving anthocyanins at a high drying temperature (60 °C).

Recently, Kaba et al. (2025) [24] reported that foam-mat drying (10% EA, 1% carboxymethylcellulose, and 5 maltodextrin) of *Cornelian cherry* purée at 60 and 70 °C helped preserve thermolabile bioactive compounds (anthocyanins) in the plant matrix. These results are in line with the obtained results, where samples containing 5% EA were dried at 60 °C. According to Table S10, the highest values of cyanidin-3-O-glucoside and pelargonidin-3-glucoside, quantified by HPLC analysis, were obtained for sample P 60_5_CD, demonstrating that the foaming agent (EA) significantly affected the content of bioactive compounds in the powders, in agreement with previous studies [8,24].

For IR drying, TAC values were significantly lower than those obtained through CD, ranging from 2.80 ± 0.46 to 62.42 ± 2.58 mg C3G·100 g^−1^ DW (Table 3). Although slight protection of anthocyanins was observed at 5% EA compared to the sample without EA, this effect was not statistically significant (Table 3). Yikilkan et al. (2025) [28] found that, when using 10% albumin, 1% carboxymethylcellulose, and 12% maltodextrin in hot air convective drying of black chokeberry pulp, anthocyanin preservation was highest at a lower temperature (50 °C) compared to higher temperatures (60 and 70 °C). These findings are in accordance with the present study, in which sample P50_10_CD exhibited the highest TAC value compared to the other powders dried in a foam-mat layer at different temperatures and EA concentrations.

Anthocyanins are a class of bioactive compounds influenced by several factors during foam-mat drying, including drying technique, temperature, foaming agent type and concentration, and foam layer thickness [8,29]. According to the HPLC analysis of extracts obtained from powders dried at 60 °C with and without EA (Table S10) using both drying techniques, 5% EA via CD yielded the highest total anthocyanin preservation (3.03 mg·100 g^−1^ DW), whereas for IR, the highest value was observed for the sample without EA, followed by the sample containing 10% EA.

The use of EA as a protective agent for bioactive compounds during the drying of blackthorn purée had a strong impact on the TPC of powders dried by CD (7.05 ± 0.11 and 10.55 ± 0.04 mg GAE·g^−1^ DW) and IR (6.96 ± 0.05 and 10.72 ± 0.09 mg GAE·g^−1^ DW), although these values remained below that of the fresh sample (14.68 ± 0.11 mg GAE·g^−1^ DW) (Table 3). Applying a higher drying temperature (70 °C) and shorter drying time in both drying techniques resulted in increased polyphenol values of 10.55 ± 0.04 (CD) and 10.72 ± 0.09 mg GAE/g DW (IR). Similar results were reported by Bayram (2024) [4], who found that convective drying of *Prunus spinosa* extract produced higher polyphenol content (14.6 ± 0.02 mg GAE·g^−1^) compared to foam-mat drying using 5% foaming agent (10.7 ± 0.02 mg GAE·g^−1^).

As shown in Table 3, increasing EA concentration from 5% to 10% led to a reduction in TPC value for all drying temperatures applied in both CD and IR, which was also observed for certain compounds quantified by HPLC (Table 6 and Table S10). Similar results were reported by Sifat et al. (2021) [30], who dried plum pulp (*Prunus domestica*) by hot air foam-mat drying at 65 °C. The authors reported that, at a foaming time of 5 min (similar to the time used for albumen foaming), an increase in albumin concentration from 4% to 6% led to a decrease in polyphenol yield from 11.71 to 10.80 mg GAE·g^−1^, with a percentage of 7.79%, while in the present study a reduction of 2.49% at 60 °C was achieved (Table 3). Thuy et al. (2022) [11] reported that the highest polyphenol values occurred at 70 °C across foam thicknesses of 0.5–1.5 cm, highlighting that the reduction in polyphenol yield may be due to polyphenol–protein interaction.

For IR drying, the TPC values decreased with increasing temperature and EA, which can be explained by possible interactions between polyphenols and the protein matrix of the foaming agent, as well as oxidative and thermal degradation processes of heat-sensitive compounds [23]. The polyphenolic profile of the extracts (Table 3) indicated that foam-mat drying did not protect these compounds as was expected. This may be due to the absence of a stabilizer influencing EA stability during drying, or to excessive EA leading to the degradation of polyphenolic compounds because of the incorporation of an excessive amount of air or the reactions that took place between these matrices. HPLC results revealed this downward trend in polyphenols, particularly in flavanols, phenolic acids, and flavonols in CD powders, and flavanols in IR powders (Table S10).

The total flavonoid content (TFC) exhibited a decrease with increasing EA concentration for both drying techniques, indicating the absence of protection of these compounds. Table 3 shows that for CD and IR, respectively, the highest TFC values when using 5% EA were obtained at 50 °C with a value of 0.59 mg EQ·g^−1^ DW for P 50_5_CD and 0.66 mg QE·g^−1^ DW for P 70_5_IR, respectively. Pearson correlation analysis indicated that the foaming agent reduced TFC, with correlation coefficients of *r* = −0.739 (CD) (Table S3) and *r* = −0.707 (IR) (Table S4).

Sifat et al. (2021) [30] reported that 6% albumin and a 5 min foaming time ensured better flavonoid protection (0.92 mg EQ·g^−1^), compared with 4% albumin (0.64 mg EQ·g^−1^). In the present study, for both drying methods (Table 3), this protection was not observed for either EA concentration (5 and 10%). Chromatographic analysis (Table S10) of the extracts obtained from powders dried at 60 °C with 0, 5, and 10% albumin showed a 21.87% decrease in flavanols and a 71.86% decrease in flavonols for CD, while IR drying resulted in a 10.76% decrease in flavanols when performing a comparative analysis between the sample with 10% EA and the control sample (without EA). This low yield of polyphenols following foam-mat drying may be attributed to the amount and type of foaming agent and to potential matrix interactions [26].

The antioxidant activity (DPPH) of the extracts obtained from CD-dried powders ranged from 25.92 ± 0.19 to 37.88 ± 0.13 µmol Trolox·g^−1^ DW, while for IR-dried powders, the values ranged between 24.40 ± 0.32 and 38.11 ± 0.28 µmol Trolox·g^−1^ DW. Pearson’s correlation showed that TPC, followed by TFC and TAC, contributed most to antioxidant activity, with *r* values of 0.875, 0.832, and 0.669 for CD, according to Table S3, and 0.900, 0.876 and 0.459 for IR, according to Table S4.

Bayram (2024) [4] also reported that foam-mat drying of *Prunus spinosa* extracts yielded polyphenol-rich powders that were correlated with high antioxidant activity, similar to our study. Ershadfarkar et al. (2023) [13] found that increasing infrared power from 400 W to 800 W significantly reduced the antioxidant activity and anthocyanin content in foam-mat dried black raspberry powders, while the amount of polyphenols increased, without a correlation between DPPH and TPC values. The increase in TPC can be explained by the possible interactions of Maillard compounds with the Folin–Ciocâlteu reagent. Tables S3 and S4 show that the antioxidant activity (DPPH) of the extracts is directly correlated with the high polyphenol content (TPC) rather than with anthocyanins (TAC).

As summarized in Table 3, samples obtained after drying by CD and IR without EA addition exhibited the highest TAC, TPC, TFC, and DPPH, compared to those with the addition of EA.

Thus, the extracts obtained were subjected to HPLC analysis to observe how the drying method and temperature influenced the yield of polyphenolic compounds. The results indicated that higher drying temperatures promoted the degradation of bioactive compounds, with reductions of 84.31% in phenolic acids and 66.64% in anthocyanins as the temperature increased from 50 to 70 °C.

### 3.5. Effect of Independent Variables on Color Parameters

#### 3.5.1. Effect on Lightness (L*)

The effect of temperature and egg white albumen (EA) on lightness (L*) of blackthorn purée (*Prunus spinosa*) dried by hot air convection (CD) and infrared (IR) drying is presented in Table 4.

Using Response Surface Methodology (RSM) by applying a Three-Level Factorial Design, the impact of temperature (50–70 °C) and EA concentration (0–10%) on the lightness (L*) values of the obtained powders was evaluated for both drying techniques. Our experimental results fit well in a second-order polynomial model.

IR drying led to slightly higher L* values, ranging from 21.20 ± 1.66 to 24.44 ± 0.30, while powders dried by CD showed slightly lower lightness, ranging from 17.40 ± 0.15 to 25.15 ± 0.43 (Table 4).

The ANOVA results derived from the fitted second-order polynomial models showed an *F*-value of 71.51 for CD and 16.84 for IR, both statistically significant (*p* < 0.05) (Table 5). The adequacy of the experimental results to the quadratic model was confirmed by the lack of significance of Lack of Fit for both models analyzed (*p* = 0.3832 for IR and 0.2893 for CD), indicating that the two selected independent variables significantly influenced powders’ lightness (L*).

The effect of the independent variables on the response variable (L*) was explained by more than 92% of the total variation, as confirmed by the high R^2^. A good correlation and quality between the results and the experimental conditions were established by the small difference between the adjustment coefficient (Adj-R^2^) and the prediction coefficient (Pred-R^2^), as shown in Table 5.

According to Table 5, the foaming agent concentration (B), the interaction between the independent variables (AB), and B^2^ were statistically significant for both drying techniques, while temperature (A) was not statistically significant. The only difference between the two drying techniques was that the quadratic temperature term (A^2^) was not significant for IR but was significant for CD. Hossain et al. (2024) [12] reported similar results regarding the absence of statistical significance of temperature and the essential role played by albumin content on the lightness of tomato powders dried by hot air convection.

The slightly lower L* values recorded for CD powders can be explained by pronounced non-enzymatic browning reactions, caused by longer exposure of the material to lower drying temperatures, which affected pigment stability. In contrast, IR led to powders with higher lightness due to faster drying time, thus preventing the degradation processes of heat-sensitive compounds and the formation of melanoidins. Ershadfarkar et al. (2023) [13] similarly reported a lower L* value for hot air convection drying compared to IR at 400 W and 600 W infrared power for foam-mat drying of black raspberries, confirming the results obtained in our study.

(18) $$L^{*}\text{\_}CD=24.50-0.30A+2.38B-0.72AB+{1.05A}^{2}-{2.94B}^{2}$$

(19) $$L^{*}\text{\_}IR=23.91+0.30A+0.70B-0.50AB-{0.39A}^{2}-{1.02B}^{2}$$

According to Equations (18) and (19), which describe the impact of drying purée by CD and IR on L*, the EA concentration (B) had a positive influence on the lightness (L*) of the powders. This effect is indicated by the high coefficient of the term in the regression equations and supported by the *F*-value of this term (Table 5). In contrast, the quadratic temperature (A^2^) and the quadratic EA concentration (B^2^) exhibited negative effects on the dependent variable (L*). However, Hossain et al. (2024) [12] reported the opposite influence of the quadratic terms of the independent variables (A^2^ and B^2^), the authors indicating a positive effect on L*, in contrast to our study.

The quadratic effect of temperature (A^2^) on L* shows that high temperatures during foam-mat drying promote the Maillard reaction, with the formation of colored specific compounds (melanoidins). These reactions are a result of the interaction between peptides and amino acids produced by the degradation of EA and naturally occurring reducing sugars in the blackthorn purée.

As shown in Table 4, increasing the drying temperature to 60 °C for the samples with 5% EA led to an increase in brightness of 1.09% for CD and 0.55% for IR, followed by a decrease at 70 °C of 6.11% for CD and 2.53% for IR. This decrease was associated with the acceleration of Maillard reactions or oxidative processes affecting pigments. Similar results were reported by Paiva et al. (2023) [31], who applied foam-mat drying at 50, 60, 70, and 80 °C to blended tropical fruit purée (acerola, guava, and pitanga) using egg albumin (6%), with the addition of Arabic gum (1%) as a foaming agent. The authors observed an increase in lightness of 18.75% when the drying temperature was raised to 60 °C, followed by a 2.72% reduction when the temperature increased to 70 °C.

**Figure 5:**
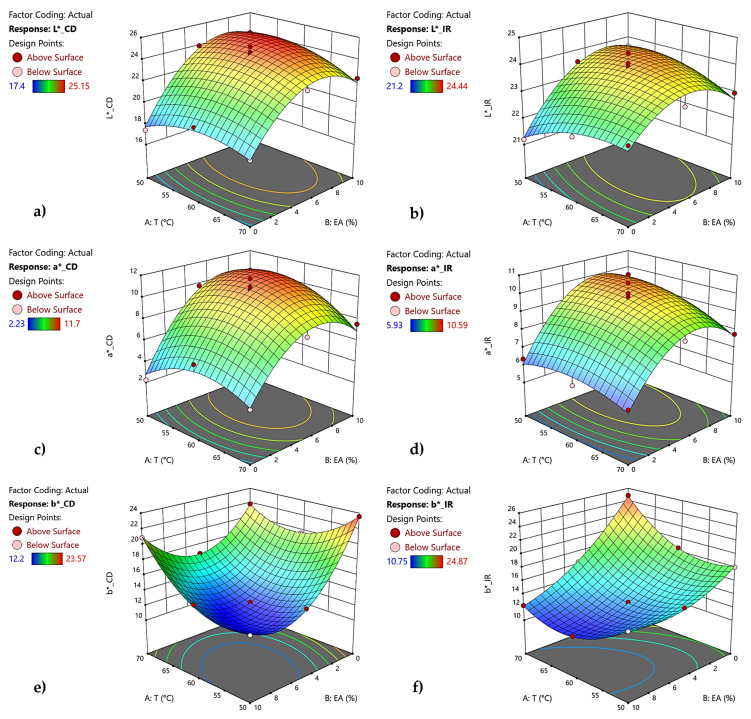
3D response surface diagrams showing the effect of the independent variables, temperature (T, °C) and egg albumin concentration (EA, %), on the response CILAB parameters for CD (**a**,**c**,**e**) and IR (**b**,**d**,**f**).

Figure 5a shows the interaction effect between the two factors on the powders obtained by CD. It can be observed that the addition of 5% foaming agent resulted in the highest L* values, with an increase ranging from 22.65 to 37.53% for all temperatures. However, Figure 5a,b indicates a slight reduction in the dependent variable, especially at a temperature of 60 °C at a 10% EA concentration increase. These results agree with those reported by Paiva et al. (2023) [31] and Hossain et al. (2024) [12].

#### 3.5.2. Effect on Redness (a*)

Table 4 shows that, for both drying techniques (CD and IR), the experimental data fitted well to a quadratic mathematical model, with statistically significant *F*-test values of 35.29 for CD and 20.92 for IR. The correlation between the redness parameter (a*) and the independent variables, as well as the overall quality of the experimental data, is reflected by the Lack of Fit value and R^2^, Adj-R^2^, and Pred-R^2^ (Table 5). According to the ANOVA results for CD, temperature (A) was not statistically significant (*p* = 0.1568), whereas the other analyzed independent variables (B, AB, A^2^, B^2^) significantly influenced the variation in the a* value of the powders. For the IR drying model (Table 5), temperature (A), the interaction between temperature and EA concentration (AB), and the quadratic temperature term (A^2^) were not statistically significant. Egg albumin (EA) concentration (B) and its quadratic term (B^2^) had a significant influence on the dependent variable (a*), with values ranging between 5.93 and 10.59, while for CD, the range of values was slightly narrower, between 2.23 and 11.70 (Table 4).

(20) $$a^{*}\text{\_}CD=10.69-0.49A+2.56B-0.95AB-{1.69A}^{2}-{3.22B}^{2}$$

(21) $$a^{*}\text{\_}IR=9.71+0.50A+1.29B-0.34AB-{0.55A}^{2}-{1.97B}^{2}$$

According to regression Equations (20) and (21), for both studied drying techniques, the strongest negative effect was observed for the quadratic EA concentration (B^2^), followed by the quadratic temperature term (A^2^), while the EA concentration (B) has the strongest positive effect on a*. A similar impact on a* produced by independent variables was reported by Hossain et al. (2024) [12] in the optimization of foam-mat drying of tomato using RSM. The authors reported similar values regarding the positive effect of albumin concentration, the negative effect of temperature, and the square of albumin concentration. However, the authors reported that the interaction between temperature and albumin content had a positive effect, while in the present study, it had a negative effect.

As shown in Table 4, the most positive effect on the a* value was produced by the independent variable at a 60 °C drying temperature, with an increase in redness (a*) values of 109.84% for CD and 22.14% for IR at 5% EA addition, followed by a decrease to 22.14% for CD and 17.94% for IR at 10% EA. This increase in the a* parameter can be associated with the protective role of the foaming agent (EA), which helps preserve anthocyanins, which are bioactive compounds with low thermal stability, especially under elevated temperature conditions, as also confirmed by liquid chromatographic analysis of the extracts (Table S10). Similarly, Tan & Sulaiman (2020) [32] reported that the addition of egg white albumen as a foaming agent in the foam-mat drying of *Hibiscus sabdariffa* L. led to an 18.11% increase in the a* value compared to powder without egg albumin addition, dried at 50 °C, and a 77.90% increase compared to the fresh sample.

The protective effect of anthocyanins observed in powder after drying of *Prunus spinosa* purée at 60 °C with 0%, 5% and 10% EA addition was confirmed by HPLC analysis, presented in Table S10. Also, the TAC of the extracts showed the highest values were achieved at a concentration of 5% EA for CD (14.74 ± 0.24 mg C3G·g^−1^ DW) and IR (11.93 ± 0.20 mg C3G·g^−1^ DW) (Table 3). According to the HPLC results, the extracts from powders obtained by CD showed the highest yield of anthocyanins at 5% EA addition, where the total quantified anthocyanins content reached 3.30 mg·100 g^−1^ DW. In this sample, cyanidin-3-O-glucoside (kuromanin) was the best-preserved compound (1.28 mg·100 g^−1^ DW), followed by pelargonidin-3-O-glucoside (callipsthein) with a value of 0.96 mg·100 g^−1^ DW, compared to the sample with 0% and 10% EA addition (Table S10).

In the case of powders dried by IR, at 10% EA concentration, cyanidin-3-O-glucoside (kuromanin) and peonidin-3-O-glucoside (callipsthein) were quantified in the highest amounts compared to the extracts with 0% and 10% EA addition (Table S10). Paiva et al. (2023) [31] also reported that, at 60 °C, anthocyanin stability during foam-mat drying in a thin layer with 6% albumin and 1% stabilizer (gum arabic, guar gum, and gelatin, respectively) was higher than at 50, 70, and 80 °C.

Pearson’s correlation analysis for both drying techniques revealed a statistically significant positive correlation between EA concentration and the color parameter a* (*r* = 0.730 for CD and *r* = 0.601 for IR). However, the correlations between TAC and EA, as well as between TAC and a*, were negative (Tables S3 and S4). This suggests that the redness (a*) can be influenced by Maillard color compounds, and the application of inadequate thermal treatments or low foaming agent stability may lead to reduced pigment preservation. Additionally, based on the results reported by other authors regarding color parameter and TAC, it was observed that although a* values were higher at 50 °C and 70 °C, the anthocyanin yield was reduced. This indicates that thermal, non-enzymatic, or oxidative degradation reactions affect the natural color compounds in the purée [11].

Foam mat drying using egg white albumen as a foaming agent under infrared drying resulted in significantly reduced values compared to samples without egg white, indicating that the application of this drying technique leads to enhanced thermal degradation of fruit pigments, particularly anthocyanins, in agreement with the results reported by Ershadfarkar et al. (2023) [13].

#### 3.5.3. Effect on Yellowness (b*)

The b* parameter value for foam-mat drying by CD of blackthorn purée using different concentrations of EA ranged from 12.20 ± 0.10 to 23.57 ± 0.15, while for IR-dried powder, the value ranged from 10.75 ± 0.27 to 24.87 ± 0.06 under the experimental conditions of temperature (A) and egg albumin addition (B), as presented in Table 4.

The experimental results obtained for this color parameter (b*), based on the experimental matrix designed through RSM for CD and IR, were successfully fitted by a second-order polynomial model. The Fisher test value indicated high statistical significance (*p* < 0.0001), while models’ adequacy and the correlation between the experimental and predicted data were confirmed by a non-significant Lack of Fit, the high R^2^, which explained more than 98% of the variation in the response and the good predictive accuracy provided by the small difference (<0.2) between Adj-R^2^ and Pred-R^2^.

Model terms were considered statistically significant when they exhibited a high *F*-value and a *p*-value below an α value of 0.05. For both models studied (CD and IR), all terms were found to be significant, as shown in Table 5.

(22) $$b^{*}\text{\_}CD=12.35+1.35A-2.62B+2.25AB+{3.02A}^{2}+{4.66B}^{2}$$

(23) $$b^{*}\text{\_}IR=12.66+0.97A-3.98B-2.29AB+{2.91A}^{2}+{1.95B}^{2}$$

Equations (22) and (23) describe the influence of the independent variable on the response (b*) and the intensity of their effects, as indicated by the coefficient value associated with the F-value (Table 5). For CD, the positive effects of the independent variables followed the intensity order B^2^ > A^2^ > AB > A, while for IR, the order was A^2^ followed by B^2^. Hossain et al. (2024) [12] reported that using RSM and applying a Box–Behnken design with three independent variables for the foam-mat drying of tomato juice, higher concentrations of egg albumin (5%) led to a 107.49% increase in the b* color parameter when the concentration of carboxymethylcellulose (CMC) was maintained at 1% and the drying temperature at 70 °C.

As illustrated in Figure 5, increasing the EA concentration up to 5% at 70 °C resulted in a 23.11% decrease in the b*value, whereas a 10% EA addition increased the b* value by 24.56%. This effect may be associated with non-enzymatic interactions between the two food matrices, which lead to the formation of powders with a reddish-brown hue, while the overall color lightness is due to the egg white albumen.

For IR-dried powders, the interaction between the independent variables exhibited a different trend compared to CD. As shown in Figure 5 and Table 4, at a lower drying temperature (50 °C), increasing EA addition to 5% and 10% decreased the b* value by 17.67% and 1.41%, respectively. Moreover, as the temperature increased, the studied response (b*) decreased more with increasing foaming agent concentration.

These variations in the b* value can be attributed to differences in the drying methods applied to the food matrix [13,14], the thickness of the drying layer, the concentration of egg albumin, the additives used as stabilizers of foaming agent [4,31] and the thermal stability of the compounds responsible for the color of the powders [8,12]. High b* values are generally associated with the formation of brown pigments resulting from two main phenomena: non-enzymatic reactions between reducing sugars from the fruit matrix and mechanisms affecting the molecular stability of anthocyanins (oxidation, cleavage, and polymerization) during high-temperature treatments, leading to the formation of brown pigments [8,29].

#### 3.5.4. Effect on Total Color Difference (∆E)

Another important parameter that influences the visual perception of consumers is the total color difference (∆E), which reflects how the experimental conditions affect the color attributes of the powders. Table 4 presents the experimental designs developed by applying a Three-Level Factorial Design and the analyzed response values (∆E) obtained under the experimental conditions (A and B).

Both models showed a high *F*-value (243.01 for CD and 131.76 for IR), indicating strong statistical significance (*p* < 0.05). The experimental results (Table 5) fitted a quadratic mathematical model, where the relationship between dependent and independent variables was explained in more than 99% of cases. The model adequacy was further confirmed by the *p*-values of 0.3291 and 0.4906 for Lack of Fit, according to Table 5. All model terms showed *p*-values below 0.05 for both drying techniques, according to Table 5.

**Figure 6:**
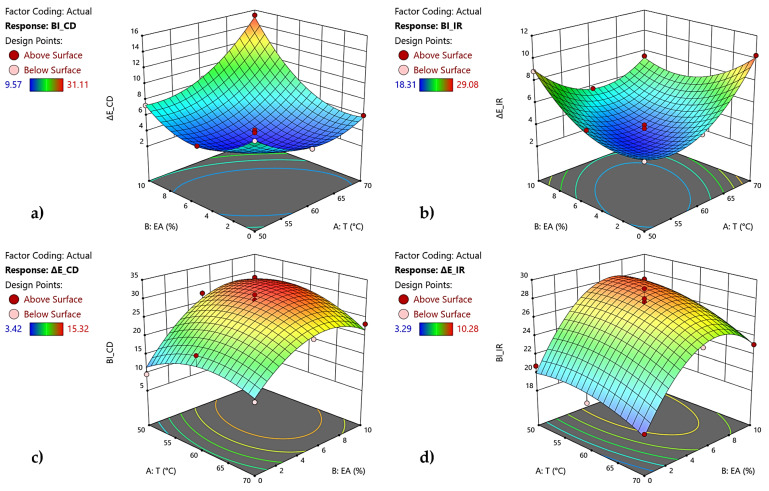
3D response surface diagrams showing the effect of the independent variables, temperature (T, °C) and egg albumin concentration (EA, %), on the response ∆E (**a**,**b**) and BI (**c**,**d**) for both drying methods.

The significance of the independent variables identified for CD drying is consistent with the findings of Brar et al. (2020) [33], with the expectation of a higher influence of temperature compared to the protein isolate concentration observed in the foam-mat drying of peach purée. These differences, indicated by F-values, highlight the stronger thermal effect in the current study. As illustrated in Figure 6a,b, the influence of the independent variables was opposite in direction, suggesting that the selected foaming agent can significantly affect the ∆E value [11,33].

(24) $$∆E\text{\_}CD=3.67+1.36A+2.14B+2.51AB+{2.37A}^{2}+{3.04B}^{2}$$

(25) $$∆E\text{\_}IR=3.53+0.92A+0.37B-1.75AB+{2.44A}^{2}+{1.85B}^{2}$$

These regression Equations (24) and (25) explain how each variable and their interaction influence the dependent variable (∆E), while the value of the coefficients and their associated *F*-value (Table 5) indicate the intensity of the effect produced on the response. For both drying techniques applied, the interaction between temperature and egg white albumen concentration (AB) exhibited different effects, as shown in Figure 6.

The interaction between the two independent variables led to an increase in ∆E, as observed in Figure 6a, where at 60 °C and 5% EA, the response value reached 3.42 ± 0.04. However, with increasing temperatures and EA concentration, the ∆E value increased rapidly by 347.95%, indicating that higher temperature coupled with a higher EA concentration leads to an accelerated non-enzymatic browning reaction, resulting in powders with different colors.

According to Figure 6b, when IR drying was applied, the interaction between temperature and foaming agent produced an opposite effect, leading to a decrease in ∆E compared with CD. Under the same experimental conditions used for CD, the ∆E value decreased by 36.69%.

For CD, it was observed that increasing the temperature up to 50 °C and 5% EA results in a 44.11% decrease in ∆E, followed by a 54.43% increase at 10% EA. Brar et al. (2020) [33] reported a similar trend for the optimization of foam-mat drying of peach purée by applying RSM, using soy protein isolate as the foaming agent. In contrast, Ershadfarkar et al. (2023) [13] observed an increase in ∆E using an intermediate infrared power (600 W) in the foam drying of black raspberry, compared to low (400 W) and higher (800 W) infrared powers, using EA as foam agent and CMC as stabilizer. However, the authors reported lower ∆E values, suggesting that heat-sensitive compounds, upon degradation, contribute to the high response values observed.

#### 3.5.5. Effect on the Browning Index (BI)

The second-order polynomial models for BI showed a *p* < 0.05 of the *F*-value for both drying techniques (Table 5). The browning index (BI) is an essential indicator of how drying conditions influence the sensory attributes of powders and reflects the relationship between processing and the availability of bioactive compounds, such as anthocyanins.

The experimental plan obtained for drying the blackthorn purée was explained with an accuracy of 94.51% for the CD model, while for IR, it was 94.66%. The ANOVA indicated that, for CD, temperature (A) and its interaction with EA concentration (AB) were not statistically significant, while for IR, the interactions between the two independent variables (AB) and the quadratic temperature term (A^2^) were non-significant.

(26) $$BI\text{\_}CD=29.50-0.69A+5.78B-2.28AB-3.99A^{2}-{6.63B}^{2}$$

(27) $$BI\text{\_}IR=27.44-1.33A+2.90B-0.39AB-{1.06A}^{2}-{4.48B}^{2}$$

According to the regression Equations (26) and (27), the EA content (B) showed the highest positive effect, while the quadratic EA term (B^2^) showed the strongest negative effect for both drying methods.

As shown in Figure 6a, in CD, increasing the EA concentration by 5% and raising the temperature to 60 °C increased the response value (BI), mainly due to the degradation of heat-sensitive compounds, oxidation reactions, and enzymatic and non-enzymatic reactions occurring during drying [23]. However, comparing powders with 5% and 10% EA, a decrease in BI was observed, as shown in the 2D and 3D representations (Figure 6). This suggests that the foaming agent partially helped preserve pigmented compounds at high temperatures and shorter drying times by reducing enzymatic and non-enzymatic reactions, as observed especially for cyanidin-3-oglucoside yield in IR drying (Table S10).

The interaction between A and B on the BI value revealed that control powders presented the lowest BI values, followed by the powders with 10% EA. At constant foaming agent concentration (10%), increasing the temperature from 50 to 70 °C reduced melanoid compound formation, resulting in a decrease in BI from 28.22 ± 0.04 to 23. 34 ± 0.30 for CD and from 27.10 ± 3.73 to 23.11 ± 1.87 for IR. These results are in agreement with Bahriye et al. (2023) [34] and Ershadfarkar et al. (2023) [13].

Pearson correlation between TAC and BI showed a negative correlation (*r* = −0.728 for CD and *r* = −0.595 for IR), while the correlation between EA and BI was positive (*r* = 0.739 for CD and *r* = 0.719 for IR), indicating that anthocyanin degradation and the formation of brown Maillard compounds contributed to increased BI values in both drying methods.

### 3.6. Identification and Validation of Optimal Drying Conditions

To optimize drying conditions (temperature and EA concentration) for both drying methods and obtain powders with coloration closest to fresh *Prunus spinosa* fruits, constraints were applied to five dependent variables (Figure S3).

Fresh *Prunus spinosa* purée had the following color parameters: L* = 18.23 ± 0.29, a* = 4.45 ± 0.10, b* = 15.87 ± 0.14, BI = 17.18 ± 0.39. Thus, in Figure S3, the constraints of the dependent variables were selected to obtain a powder with color characteristics as close as possible to the control sample (fresh purée). In Figure S3, for both drying techniques, the minimum points of the independent variables (red dots) represent the experimental conditions that lead to obtaining a powder like the control sample; these are indicated on the ramp graphs with blue dots.

For CD, 10 solutions were identified (Table S5) with desirability values between 0.166 and 0.953, while for IR, 13 solutions (Table S6) were identified with desirability values between 0.257 and 0.897. The most suitable experimental conditions used to validate the solution were those that presented the desirability value closest to 1. Following the selection of the constraints (Figure S3) of the dependent variables, for CD, a temperature of 50 °C and 0% EA led to the desired result, while for IR, a temperature of 70 °C and 0% EA was identified.

Drying under these optimized conditions resulted in powders with color parameter values within the predicted (CI = 95%) ranges for both drying methods (Table S7).

### 3.7. Principal Component Analysis (PCA)

PCA was applied to assess the physicochemical, functional and colorimetric characteristics of the powders. The indicators used in drying were temperature (T); foaming agent concentration (EA); moisture effective diffusivity (D_eff_); drying period (DT); final moisture content of the powders (DW); color parameters of brightness (L*), redness/blueness (a*) yellowness/greenness (b*), total color difference (∆E), and brown index (BI); powder characteristics of bulk density (BD), tapped density (TD), Carr Index (CI), and Hausner Ratio (HR); and phytochemical parameters of total anthocyanin content (TAC), total polyphenol content (TPC), total flavonoid content (TFC) and antioxidant activity (DPPH).

The dimensionality of the analyzed indicators was performed using the PCA method, selecting the principal components (PCs) that contributed most to the variation in the response. As shown in Table S8, the first two PCs explained 62.9% of the total variation, while inclusion of PC3 and PC4 increased the cumulative variance explained to 87.5%, all presenting eigenvalues > 1 [15]. Gao et al. (2022) [10] reported that two principal components were necessary to explain 84.57% of the physicochemical variability in microwave-assisted foam-mat drying of blueberry pulp, while Widhanti et al. (2024) [35] found that the first two PCs explained 60.4% of the variation in the physicochemical properties and antioxidant activity of *Physalis angulata* L. foam-dried powders enriched with *Moringa oleifera* L.

**Figure 7:**
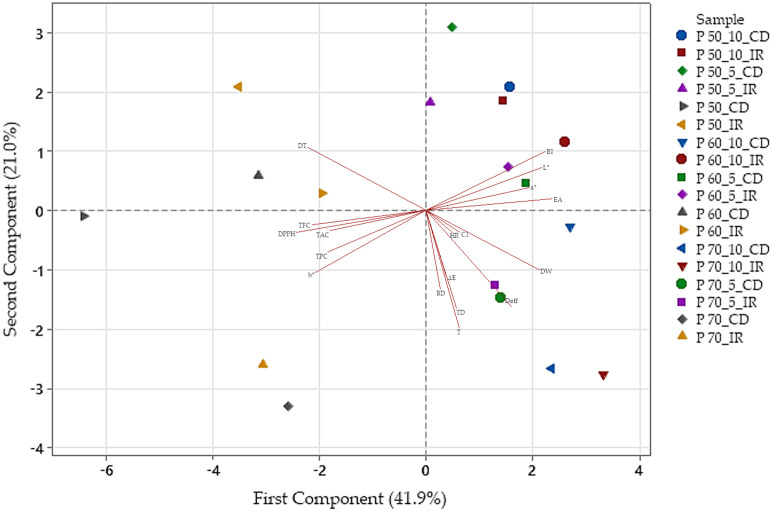
PCA score and loading plots showing the clustering and variation of powder samples based on the first two principal components (PCs).

Figure 7 shows that PC1 correlated positively with EA, L*, a*, BI, and DW and negatively correlated with TAC, TPC, TFC, and DPPH, while PC2 was positively correlated with BI and negatively correlated with ∆E. Gao et al. (2022) [10] obtained similar results regarding the correlation of BI and ∆E associated with PC2, respectively, that the load of PC1 highlighted changes in the physicochemical characteristics of the powders, such as porosity and colorimetric L*, a*, b* values.

A separation between powders dried with and without foaming agent was observed. The first quadrant groups powders dried at 50 and 60 °C without EA by CD and IR. The second quadrant contains powders dried at 50 °C with 5% EA, 50 °C with 10% EA, and 60 °C with 5% EA by CD and IR; and powder dried at 60 °C with 10% EA by IR—this quadrant being associated with high BI, L*, and a* values. The third quadrant included powders dried at 70 °C with 5% and 10% EA using both drying techniques, and powder dried at 60 °C with 10% EA using CD, which was associated with the highest values of dry matter (DW) and effective moisture diffusivity (D_eff_), while the last quadrant included powders dried at 70 °C using both techniques, with the highest values of CTP and b*.

High TFC, TAC, and DPPH values were located between the first and last quadrants, suggesting that powders dried at 70 °C exhibited high antioxidant activity, similar to the study conducted by Widhanti et al. (2024) [35].

### 3.8. HPLC Analysis of Optimized Powders

HPLC analysis was performed on extracts obtained from powders produced under optimized drying conditions without foaming agent, based on the results obtained from PCA analysis and optimization of the mathematical modeling of color parameters, as they presented the highest amounts of bioactive compounds.

Table 6 shows that the polyphenolic profile varied, confirming that the temperature and drying technique used significantly influenced the preservation of bioactive compounds. CD-dried powders contained high amounts of ellagic acid, quercetin 3-diglucoside, epigallocatechin, catechin, naringin, peonidin 3-O-glucoside, kuromanin, and callistephin at all temperatures compared with the IR-dried sample, highlighting the protective effect of hot air drying on bioactive compounds.

Powder dried by CD at 50 °C without EA showed the highest total quantified phenolic content (943.93 mg·100 g^−1^ DW), while the IR-dried powder at 70 °C showed the highest content of quantified compounds (776.43 mg·100 g^−1^ DW), consistent with the color optimization results.

Nistor et al. (2023) [2] identified similar phenolic acids and flavonoids (gallic acid, chlorogenic acid, caffeic acid, vanillic acid, *p*-coumaric acid, flavonols: myricetin and isorhamnetin, epicatechin, catechin), and anthocyanins (delphinidin 3-O-β-D-glucoside, cyanidin 3-O-glucoside, cyanidin 3-O-rutinoside, peonidin 3-O-glucoside) in freeze-dried peels of *Prunus spinosa*.

Table 6 shows that the phenolic compounds in blackthorn powder that were predominantly present in the highest quantities were gallic acid, epigallocatechin, and catechin, which are reported in the literature as being present in fruits and having high bioactive properties [2,35].

## 4. Conclusions

Harnessing natural resources offers a sustainable alternative to developing food matrices appreciated by consumers, especially when the powders used are rich in bioactive compounds.

In this study, hot air (CD) and infrared (IR) drying methods were evaluated for their influence on the color parameters and polyphenolic profile of wild blackthorn purée, both in native form and with added EA (5% and 10%) at different temperatures. The Midilli and Page models provide the best fit for the experimental drying data, with higher D_eff_ values for IR, explained by the accelerated rate of moisture removal from the vegetable matrix.

Optimization using a Three-Level Experimental Design identified and validated 50 °C and 0% EA for CD and 70 °C and 0% for IR, respectively, as optimal conditions. These conditions yielded powders with the lowest BI and provided the highest quantified polyphenolic content according to HPLC analysis. CD drying at 60 °C with 5% EA provided a slight improvement in anthocyanin level (6.81%), with higher values of kuromanin and callistephin. In contrast, IR drying showed lower values (1.02%) under the same formulation conditions, although some glycosidic forms of peonidin and cyanidin were better preserved at 10% EA.

Egg white albumen as a foaming agent did not confer substantial protection of bioactive compounds, as expected. This can be attributed to numerous physicochemical processes, such as the interaction between the compounds in the purée and the protein matrix (Maillard reaction), and the low stability of the foaming agent during drying, which led to the collapse of the incorporated air.

Drying temperature significantly affected the color of the powders, mainly through its influence on the total anthocyanin content, which is responsible for the final powder color, highlighting the reduced stability of these pigments at higher drying temperatures.

However, the results should be interpreted as specific to the batch investigated, as the fruits were collected from a single location and season, which may limit the generalizability of the findings.

Future research will focus on incorporating the obtained powders into complex food matrices to assess consumer acceptance of products containing these fruits and the potential application of these powders to achieve a food product with high value provided by a complex profile of polyphenolic compounds with high antioxidant activity.

## Figures and Tables

**Table 1:** Effective moisture diffusion (D_eff_) and energy activation (E_a_) associated with the determination coefficient (R^2^) for the different drying methods (CD and IR) applied to blackthorn purée with 0%, 5% and 10% EA at 50, 60 and 70 °C.

Sample Code	Drying Method	T, °C	EA, %	D_eff_ × 10^−9^, m^2^·s^−1^	R^2^	E_a_, kJ·mol^−1^	R^2^
P 50_CD	CD	50	0	0.76 ± 0.050 ^c, B^	0.9406	22.30 ± 0.72 ^a, A^	0.9303
P 60_CD		60	0	0.87 ± 0.06 ^b, B^	0.9455		
P 70_CD		70	0	1.23 ± 0.10 ^a, B^	0.9618		
P 50_5_CD	CD	50	5	0.81 ± 0.04 ^c, B^	0.9606	19.91 ± 1.41 ^b, A^	0.9949
P 60_5_CD		60	5	1.03 ± 0.09 ^b, AB^	0.9619		
P 70_5_CD		70	5	1.24 ± 0.091 ^a, B^	0.9198		
P 50_10_CD	CD	50	10	1.09 ± 0.06 ^c, A^	0.964	16.83 ± 1.41 ^c, A^	0.9095
P 60_10_CD		60	10	1.19 ± 0.03 ^b, A^	0.9672		
P 70_10_CD		70	10	1.57 ± 0.07 ^a, A^	0.9787		
P 50_IR	IR	50	0	0.80 ± 0.06 ^c, B^	0.9521	20.65 ± 0.43 ^a, B^	0.9939
P 60_IR		60	0	0.98 ± 0.04 ^b, B^	0.9859		
P 70_IR		70	0	1.25 ± 0.09 ^a, B^	0.9625		
P 50_5_IR	IR	50	5	0.87 ± 0.09 ^c, B, B^	0.9709	19.68 ± 0.74 ^b, B^	0.9841
P 60_5_IR		60	5	1.03 ± 0.06 ^b, B^	0.9723		
P 70_5_IR		70	5	1.34 ± 0.11 ^a, B^	0.9616		
P 50_10_IR	IR	50	10	1.11 ± 0.05 ^c, A^	0.9394	15.30 ± 0.70 ^c, B^	0.9582
P 60_10_IR		60	10	1.23 ± 0.08 ^b, A^	0.9385		
P 70_10_IR		70	10	1.65 ± 0.04 ^a, A^	0.9381		

Note: Different lowercase letters (a, b, c) indicate statistically significant differences between EA concentrations at the same temperature within each drying method. Different uppercase letters (A, B) indicate statistically significant differences between temperatures and drying methods at the same EA concentration, based on ANOVA followed by Tukey’s test (*p* < 0.05).

**Table 2:** Flowability properties of CD-dried and IR-dried wild *Prunus spinosa* powder.

Sample Code	Drying Method	T, °C	EA, %	Bulk Density, g/mL	Tapped Density, g/mL	Carr’s Index, %	Hausner Ratio, %
P 50_CD	CD	50	0	0.566 ± 0.011 ^a, A^	0.703 ± 0.010 ^a, B^	19.49 ± 0.78 ^b, B^	1.24 ± 0.01 ^b, B^
P 60_CD		60	0	0.574 ± 0.017 ^a, A^	0.709 ± 0.020 ^b, B^	19.07 ± 2.86 ^b, B^	1.24 ± 0.04 ^b, B^
P 70_CD		70	0	0.570 ± 0.006 ^a, A^	0.776 ± 0.012 ^a, A^	26.57 ± 1.66 ^a, A^	1.36 ± 0.03 ^a, A^
P 50_5_CD	CD	50	5	0.471 ± 0.011 ^c, B^	0.648 ± 0.008 ^b, B^	27.20 ± 1.80 ^a, A^	1.37 ± 0.03 ^a, A^
P 60_5_CD		60	5	0.570 ± 0.012 ^a, A^	0.770 ± 0.020 ^a, A^	25.95 ± 0.96 ^a, A^	1.35 ± 0.02 ^a, A^
P 70_5_CD		70	5	0.546 ± 0.010 ^b, A^	0.744 ± 0.011 ^ab, A^	26.66 ± 0.68 ^a, A^	1.36 ± 0.01 ^a, A^
P 50_10_CD	CD	50	10	0.509 ± 0.009 ^b, C^	0.657 ± 0.008 ^b, B^	22.58 ± 1.65 ^b, A^	1.29 ± 0.03 ^b, A^
P 60_10_CD		60	10	0.542 ± 0.006 ^b, B^	0.721 ± 0.027 ^b, A^	24.66 ± 3.49 ^a, A^	1.33 ± 0.06 ^a, A^
P 70_10_CD		70	10	0.566 ± 0.011 ^a, A^	0.757 ± 0.022 ^ab, A^	25.14 ± 2.57 ^a, A^	1.34 ± 0.05 ^ab, A^
P 50_IR	IR	50	0	0.469 ± 0.007 ^a, B^	0.643 ± 0.008 ^b, B^	27.08 ± 0.57 ^a, A^	1.37 ± 0.01 ^a, A^
P 60_IR		60	0	0.542 ± 0.011 ^b, A^	0.720 ± 0.010 ^b, A^	24.67 ± 2.30 ^a, A^	1.33 ± 0.04 ^a, A^
P 70_IR		70	0	0.523 ± 0.011 ^b, A^	0.726 ± 0.027 ^b, A^	27.92 ± 1.82 ^a, A^	1.39 ± 0.04 ^a, A^
P 50_5_IR	IR	50	5	0.518 ± 0.018 ^b, B^	0.692 ± 0.009 ^a, B^	25.25 ± 1.81^a, A^	1.34 ± 0.03 ^a, A^
P 60_5_IR		60	5	0.596 ± 0.007 ^a, A^	0.763 ± 0.011 ^a, A^	21.54 ± 1.77 ^a, A^	1.28 ± 0.03 ^a, A^
P 70_5_IR		70	5	0.566 ± 0.011 ^a, A^	0.738 ± 0.011 ^b, A^	23.26 ± 0.73 ^b, A^	1.30 ± 0.01^b, A^
P 50_10_IR	IR	50	10	0.466 ± 0.004 ^b, C^	0.634 ± 0.008 ^b, C^	26.42 ± 1.35 ^a, B^	1.36 ± 0.03 ^a, A^
P 60_10_IR		60	10	0.530 ± 0.011 ^b, B^	0.677 ± 0.009 ^c, B^	21.74 ± 2.26 ^a, A^	1.28 ± 0.04 ^a, B^
P 70_10_IR		70	10	0.601 ± 0.024 ^a, A^	0.811 ± 0.022 ^a, A^	25.97 ± 0.96 ^ab, A^	1.35 ± 0.02 ^ab, AB^

Note: Different lowercase letters (a, b, c) indicate statistically significant differences between EA concentrations at the same temperature within each drying method. Different uppercase letters (A, B, C) indicate statistically significant differences between temperatures and drying methods at the same EA concentration, based on ANOVA followed by Tukey’s test (*p* < 0.05).

**Table 3:** Phytochemicals content and antioxidant activity of fresh purée, CD-dried and IR-dried wild blackthorn powder at different temperatures and EA concentrations.

Sample Code	Drying Method	T, °C	EA, %	TAC, mg C3G·100 g^−1^ DW	TPC, mg GAE·g^−1^ DW	TFC, mg QE·g^−1^ DW	DPPH, µmol Trolox·g^−1^ DW
P Fresh	-	-	-	62.42 ± 2.58	14.68 ± 0.11	1.26 ± 0.04	78.95 ± 0.71
P 50_CD	CD	50	0	26.32 ± 1.02 ^a, A^	8.79 ± 0.06 ^a, B^	0.75 ± 0.04 ^a, A^	36.27 ± 0.13 ^a, B^
P 60_CD		60	0	15.11 ± 0.38 ^b, C^	8.23 ± 0.01 ^a, C^	0.60 ± 0.09 ^a, B^	33.19 ± 0.45 ^a, C^
P 70_CD		70	0	18.80 ± 0.59 ^a, B^	10.55 ± 0.04 ^a, A^	0.74 ± 0.01 ^a, A^	37.88 ± 0.13 ^a, A^
P 50_5_CD	CD	50	5	16.32 ± 0.91 ^b, A^	7.03 ± 0.09 ^b, C^	0.62 ± 0.03 ^b, B^	28.67 ± 0.28 ^b, AB^
P 60_5_CD		60	5	16.36 ± 0.27 ^a, A^	7.23 ± 0.07 ^b, B^	0.59 ± 0.02 ^a, A^	28.41 ± 0.26 ^b, B^
P 70_5_CD		70	5	15.90 ± 0.33 ^b, A^	7.89 ± 0.05 ^b, A^	0.59 ± 0.02 ^b, A^	29.17 ± 0.24 ^b, A^
P 50_10_CD	CD	50	10	17.94 ± 0.19 ^b, A^	7.27 ± 0.06 ^b, B^	0.66 ± 0.01 ^b, A^	29.50 ± 0.21 ^b, A^
P 60_10_CD		60	10	13.13 ± 0.38 ^c, B^	7.05 ± 0.11 ^c, B^	0.55 ± 0.01^a, B^	25.92 ± 0.19 ^c, C^
P 70_10_CD		70	10	11.57 ± 0.55 ^c, C^	8.07 ± 0.02 ^b, A^	0.57 ± 0.02 ^c, C^	27.46 ± 0.79 ^c, B^
P 50_IR	IR	50	0	22.23 ± 0.50 ^a, A^	8.15 ± 0.01 ^c, B^	0.77 ± 0.04 ^a, A^	34.31 ± 0.15 ^a, B^
P 60_IR		60	0	12.86 ± 0.21^a, C^	8.01 ± 0.02 ^a, C^	0.63 ± 0.03 ^a, B^	32.14 ± 0.21 ^a, C^
P 70_IR		70	0	18.91 ± 0.47 ^a, B^	10.72 ± 0.09 ^a, A^	0.79 ± 0.03 ^a, A^	38.11 ± 0.28 ^a, A^
P 50_5_IR	IR	50	5	4.55 ± 0.20 ^b, C^	9.56 ± 0.02 ^a, A^	0.68 ± 0.03 ^b, A^	33.51 ± 0.39 ^b, A^
P 60_5_IR		60	5	13.37 ± 0.23 ^a, B^	6.96 ± 0.05 ^b, C^	0.61 ± 0.02 ^a, B^	26.79 ± 0.07 ^b, C^
P 70_5_IR		70	5	15.16 ± 0.29 ^b, A^	7.18 ± 0.05 ^b, B^	0.70 ± 0.02 ^b, A^	28.10 ± 0.11 ^b, B^
P 50_10_IR	IR	50	10	2.80 ± 0.46 ^c, B^	9.59 ± 0.03 ^b, A^	0.70 ± 0.03 ^b, A^	32.03 ± 0.32 ^c, A^
P 60_10_IR		60	10	11.01 ± 0.52 ^b, A^	6.57 ± 0.03 ^c, C^	0.58 ± 0.01 ^b, B^	24.40 ± 0.32 ^c, C^
P 70_10_IR		70	10	9.84 ± 0.36 ^c, A^	7.43 ± 0.02 ^b, B^	0.64 ± 0.05 ^b, AB^	27.04 ± 0.55 ^c, B^

Note: Different lowercase letters (a, b, c) indicate statistically significant differences between EA concentrations at the same temperature within each drying method. Different uppercase letters (A, B, C) indicate statistically significant differences between temperatures and drying methods at the same EA concentration, based on ANOVA followed by Tukey’s test (*p* < 0.05).

**Table 4:** Three-Level Factorial Design for hot air convection (CD) and infrared (IR) drying of blackthorn purée at different temperatures and EA concentrations.

	Factor 1	Factor 2	Response 1		Response 2		Response 3		Response 4		Response 5		Response 6		Response 7	
Run	A: T, °C	B: EA, %	L*_CD	L*_IR	a*_CD	a*_IR	b*_CD	b*_IR	∆E_CD	∆E_IR	C_CD	C_IR	h_CD	h_IR	BI_CD	BI_IR
1	60	0	19.49 ± 0.12	22.06 ± 0.03	5.56 ± 0.07	5.99 ± 0.11	19.56 ± 0.06	18.62 ± 0.23	4.39 ± 0.22	4.97 ± 0.31	21.31 ± 0.05	19.56 ± 0.19	74.81 ± 0.49	72.49 ± 0.36	19.91 ± 0.16	18.99 ± 0.31
2	60	5	24.22 ± 0.10	24.44 ± 0.30	10.23 ± 0.03	10.59 ± 1.10	12.26 ± 0.10	12.44 ± 0.18	3.42 ± 0.04	4.00 ± 0.90	15.97 ± 0.05	16.36 ± 0.59	50.16 ± 0.32	49.71 ± 3.25	28.47 ± 1.08	29.08 ± 1.96
3	70	0	18.54 ± 0.16	22.52 ± 1.82	3.56 ± 0.10	5.93 ± 1.69	21.81 ± 0.20	24.87 ± 0.06	6.02 ± 0.15	10.28 ± 1.12	22.10 ± 0.18	25.60 ± 0.41	80.73 ± 0.34	76.64 ± 3.68	13.83 ± 0.23	18.31 ± 3.70
4	60	5	25.15 ± 0.23	24.07 ± 0.15	11.70 ± 0.25	10.02 ± 0.76	12.20 ± 0.10	12.62 ± 0.25	3.77 ± 0.07	3.67 ± 0.50	16.90 ± 0.05	16.14 ± 0.50	46.21 ± 0.32	51.64 ± 3.11	31.11 ± 0.05	28.04 ± 1.60
5	50	10	24.00 ± 0.02	23.65 ± 2.45	10.03 ± 0.02	9.55 ± 2.45	13.66 ± 0.13	14.70 ± 1.44	7.32 ± 0.10	8.87 ± 2.06	16.95 ± 0.11	17.66 ± 1.13	53.71 ± 0.22	57.12 ± 8.35	28.22 ± 0.04	27.10 ± 3.73
6	60	5	24.16 ± 0.21	23.70 ± 0.21	10.15 ± 0.05	9.45 ± 0.83	12.45 ± 0.25	12.81 ± 0.31	3.49 ± 0.07	3.33 ± 0.25	16.07 ± 0.05	15.92 ± 0.45	50.80 ± 0.32	53.58 ± 2.89	28.33 ± 0.08	27.01 ± 2.05
7	70	5	22.74 ± 0.06	23.13 ± 0.24	7.98 ± 0.06	8.38 ± 0.10	16.77 ± 0.38	16.38 ± 0.06	6.96 ± 0.38	6.58 ± 0.05	18.57 ± 0.29	18.40 ± 0.02	64.54 ± 0.82	62.91 ± 0.35	24.06 ± 0.26	24.78 ± 0.21
8	50	5	23.96 ± 0.01	23.57 ± 0.66	9.76 ± 0.01	9.14 ± 0.94	14.16 ± 0.09	14.91 ± 1.23	4.74 ± 0.03	5.34 ± 1.03	17.30 ± 0.07	17.63 ± 0.52	54.94 ± 0.21	57.90 ± 4.95	27.56 ± 0.82	26.32 ± 1.86
9	50	0	17.40 ± 0.15	21.20 ± 1.66	2.23 ± 0.06	6.36 ± 1.32	23.57 ± 0.15	18.11 ± 1.48	8.05 ± 0.15	4.64 ± 1.09	23.67 ± 0.14	19.24 ± 1.21	84.6 ± 0.17	70.56 ± 4.71	9.57 ± 0.16	20.76 ± 3.01
10	60	5	24.51 ± 0.17	23.72 ± 0.42	10.69 ± 0.05	9.48 ± 1.00	12.30 ± 0.10	12.75 ± 0.20	3.90 ± 0.05	3.29 ± 0.43	16.31 ± 0.05	15.88 ± 0.35	49.06 ± 0.32	53.37 ± 2.51	29.31 ± 1.27	27.05 ± 1.85
11	60	10	23.43 ± 0.02	23.38 ± 0.05	9.11 ± 0.03	8.69 ± 0.03	14.66 ± 0.15	10.75 ± 0.27	8.65 ± 0.12	5.76 ± 0.22	17.26 ± 0.14	13.83 ± 0.20	58.14 ± 0.22	51.04 ± 0.79	26.42 ± 0.06	25.28 ± 0.01
12	70	10	22.28 ± 0.09	22.97 ± 0.31	7.55 ± 0.09	7.76 ± 0.79	20.89 ± 0.10	12.29 ± 1.13	15.32 ± 0.14	7.49 ± 1.34	22.23 ± 0.07	14.57 ± 0.50	69.64 ± 0.44	57.6 ± 5.11	23.34 ± 0.30	23.11 ± 1.87
13	60	5	24.66 ± 0.05	23.95 ± 0.36	10.93 ± 0.03	9.82 ± 0.88	12.33 ± 0.10	12.56 ± 0.31	4.13 ± 0.04	3.36 ± 0.35	16.49 ± 0.05	15.95 ± 0.23	48.51 ± 0.32	52.00 ± 3.05	29.72 ± 0.95	27.69 ± 1.91

**Table 5:** ANOVA of second-order polynomial models for CIELAB parameters.

Source	L*				a*				b*				∆E				BI			
	CD		IR		CD		IR		CD		IR		CD		IR		CD		IR	
	F-Value	*p*-Value	F-Value	*p*-Value	F-Value	*p*-Value	F-Value	*p*-Value	F-Value	*p*-Value	F-Value	*p*-Value	F-Value	*p*-Value	F-Value	*p*-Value	F-Value	*p*-Value	F-Value	*p*-Value
Model	71.51	<0.0001	16.84	0.0009	35.29	<0.0001 ^a^	20.92	0.0004 ^a^	1572.13	<0.0001 ^a^	855.99	<0.0001 ^a^	243.01	<0.0001 ^a^	131.76	<0.0001 ^a^	24.10	0.0003 ^a^	24.81	0.0003 ^a^
A	2.55	0.1544 ^b^	0.0638	0.8078	2.51	0.1568 ^b^	5.53	0.0509 ^b^	413.69	<0.0001 ^a^	137.88	<0.0001 ^a^	104.76	<0.0001 ^a^	55.74	0.0001 ^a^	0.7065	0.4284 ^b^	9.28	0.0187 ^a^
B	160.44	<0.0001 ^a^	28.42	0.0011	68.91	<0.0001 ^a^	37.14	0.0005 ^a^	1567.87	<0.0001 ^a^	2317.42	<0.0001 ^a^	257.08	<0.0001 ^a^	9.16	0.0192 ^a^	50.03	0.0002 ^a^	44.27	0.0003 ^a^
AB	9.65	0.0172 ^a^	9.58	0.0175	6.38	0.0395 ^a^	1.73	0.2300 ^b^	768.18	<0.0001 ^a^	513.44	<0.0001 ^a^	235.68	<0.0001 ^a^	136.22	<0.0001 ^a^	5.22	0.0563 ^b^	0.52	0.4949 ^b^
A^2^	14.44	0.0067 ^a^	4.00	0.0856	13.82	0.0075 ^a^	3.08	0.1226 ^b^	957.47	<0.0001 ^a^	571.90	<0.0001 ^a^	145.77	<0.0001 ^a^	182.12	<0.0001 ^a^	10.96	0.0129 ^a^	2.72	0.1432 ^b^
B^2^	112.88	<0.0001 ^a^	27.46	0.0012	50.41	0.0002 ^a^	39.93	0.0004 ^a^	2284.81	<0.0001 ^a^	256.96	<0.0001 ^a^	239.70	<0.0001 ^a^	104.18	<0.0001 ^a^	30.33	0.0009 ^a^	48.37	0.0002 ^a^
Lack of Fit	1.78	0.2893 ^b^	1.33	0.3832	2.05	0.2491 ^b^	1.53	0.3367 ^b^	5.75	0.0622 ^b^	3.02	0.1565 ^b^	1.57	0.3291 ^b^	0.9667	0.4906 ^b^	6.08	0.0569 ^b^	0.23	0.2127 ^b^
R^2^	0.9808		0.9233		0.9618		0.9373		0.9991		0.9984		0.9943		0.9895		0.9451		0.9466	
Adj-R^2^	0.9671		0.8684		0.9346		0.8925		0.9985		0.9972		0.9902		0.9820		0.9059		0.9084	

Note: R^2^—coefficient of determination, Adj-R^2^—adjusted R^2^, ^a^—significant, ^b^—not significant.

**Table 6:** Chromatographic analysis of extracts obtained from control powders dried at 50 °C and 70 °C using CD and IR.

Compound, mg·100 g^−1^	50 °C		70 °C	
	CD	IR	CD	IR
Phenolic acids				
Gallic acid	54.93 ± 4.12 ^b, C^	97.91 ± 15.83 ^a, B^	n.d.	277.68 ± 8.23 ^A^
4-Hydroxybenzoic acid	5.03 ± 0.25 ^A^	n.d.	1.21 ± 0.02 ^B^	n.d.
Chlorogenic acid	n.d.	4.29 ± 0.24 ^A^	4.64 ± 0.53 ^a, A^	4.59 ± 0.08 ^a, A^
Ellagic acid	6.10 ± 1.05 ^a, A^	3.12 ± 0.06 ^b, B^	3.18 ± 0.02 ^a, B^	3.17 ± 0.01^a, B^
*p*-Coumaric acid	0.76 ± 0.01 ^a, A^	0.46 ± 0.05 ^b, B^	0.44 ± 0.08 ^B^	n.d.
Cinnamic acid	0.84 ± 0.26 ^a, A^	0.49 ± 0.10 ^b, B^	0.44 ± 0.10 ^a, B^	0.47 ± 0.07 ^a, B^
Vanillic acid	n.d.	traces	traces	n.d.
Sinapic acid	n.d.	n.d.	0.71 ± 0.12	n.d.
Syringic acid	n.d.	traces	n.d.	n.d.
Caffeic acid	n.d.	n.d.	n.d.	traces
Flavones				
Apigenin	1.05 ± 0.25	n.d.	n.d.	n.d.
Flavonols				
Quercetin 3,4′-diglucoside	n.d.	1.28 ± 0.05 ^A^	1.38 ± 0.08 ^a, A^	1.37 ± 0.01^a, A^
Quercetin 3-glucoside	8.15 ± 1.52 ^a, A^	2.42 ± 0.15 ^b, B^	n.d.	2.99 ± 0.07 ^B^
Rutin trihydrate	n.d.	n.d.	n.d.	1.41 ± 0.14 ^A^
Flavanols				
Epigallocatechin	662.89 ± 3.16 ^a, A^	300.11 ± 9.84 ^b, C^	288.06 ± 5.52 ^a, C^	349.48 ± 9.00 ^b, B^
Catechin	193.65 ± 2.14 ^a, A^	93.02 ± 4.52 ^b, D^	106.72 ± 0.77 ^b, C^	129.44 ± 8.91^a, B^
Epicatechin gallate	n.d.	2.61 ± 0.15 ^A^	2.95 ± 0.05 ^A^	n.d.
Flavanones				
Naringin	4.40 ± 1.18 ^a, A^	3.60 ± 0.97 ^a, A^	3.54 ± 0.27 ^a, A^	3.83 ± 0.25 ^a, A^
Hesperidin	n.d.	traces	traces	n.d.
Anthocyanins				
Peonidin 3-O-glucoside	2.28 ± 0.11 ^a, A^	1.50 ± 0.02 ^b, B^	0.97 ± 0.15 ^a, C^	0.92 ± 0.03 ^a, C^
Kuromanin (Cyanidin-3-O-glucoside)	2.93 ± 0.22 ^a, A^	1.44 ± 0.06 ^b, B^	1.04 ± 0.34 ^a, B^	1.08 ± 0.05 ^a, B^
Callipsthein (Pelargonidin-3-glucoside)	0.92 ± 0.03 ^A^	traces	0.79 ± 0.16 ^A^	traces
Keracyanin (Cyanidin-3-O-galactoside)	n.d.	n.d.	n.d.	n.d.
Terpenoids				
Cafestol	n.d.	traces	n.d.	traces
Alkaloids				
Caffeine	n.d.	n.d.	n.d.	traces

Note: n.d.—not detected. Different lowercase letters indicate statistically significant differences between powders dried at the same temperature, but using different drying methods, while different uppercase letters mean statistically significant differences between powders for the same bioactive compound.

## Data Availability

The original contributions presented in this study are included in the article/Supplementary Materials. Further inquiries can be directed to the corresponding author.

## Supplementary Materials

The following supporting information can be downloaded at https://www.mdpi.com/article/10.3390/foods15142449/s1, Figure S1: Predicted versus experimental (MR) values for CD blackthorn purée with 0%, 5% and 10% EA, fitted using the Logarithmic model (a,d,g), the Page model (b,e,h) and the Midilli model (c,f,i); Figure S2: Predicted versus experimental (MR) values for IR blackthorn purée with 0%, 5% and 10% EA, fitted using the Logarithmic model (a,d,g), the Page model (b,e,h) and the Midilli model (c,f,i); Figure S3: The constraints applied to the dependent variables (blue dots) and the independent variables associated with them (red dots) for CD-dried (A) and IR-dried (B) blackthorn powders; Table S1: Mathematical models applied to the experimental data for CD drying; Table S2: Mathematical models applied to the experimental data for IR drying; Table S3: Pearson correlation for the CD-dried blackthorn powders; Table S4: Pearson correlation for the IR-dried blackthorn powders; Table S5: Solutions identified for the optimization of CD drying conditions of blackthorn purée based on color parameters; Table S6: Solutions identified for the optimization of IR drying conditions of blackthorn purée based on color parameters; Table S7: Experimental confirmation (n = 3) of optimal independent variable for CD and IR drying methods; Table S8: Eigenvalue analysis of the correlation matrix calculated for the dependent variables; Table S9: Eigenvectors corresponding to principal components with eigenvalues greater than 1; Table S10: Chromatographic analysis of extracts obtained from powders dried at 60 °C using CD and IR, with 0%, 5%, and 10% EA concentrations.
