# Supplementary material for: Optimization of Wild Blackthorn (Prunus spinosa L.) Purée Drying: Effect of Egg Albumen Concentration Using Response Surface Methodology
Source: Foods. 2026 Jul 10;15(14):2449. doi: 10.3390/foods15142449 (PMC13407606; doi:10.3390/foods15142449)
Supplement: Supplementary file 1 [file foods-15-02449-s001.zip › foods-4408328-supplementary.pdf]

Table S1. Mathematical models applied to the experimental data for CD drying

Mathematical model	T, °C	EA, %	k	n	a	b	c	RMSE	χ^2	R^2
**Lewis/ Newtown**	50	0	0.025	-	-	-	-	2.24×10^{-2}	0.54×10^{-3}	0.9928
	60	0	0.027	-	-	-	-	2.17×10^{-2}	0.51×10^{-3}	0.9934
	70	0	0.031	-	-	-	-	3.02×10^{-2}	1.00×10^{-3}	0.9892
	50	5	0.025	-	-	-	-	1.31×10^{-2}	0.19×10^{-3}	0.9977
	60	5	0.032	-	-	-	-	2.15×10^{-2}	0.50×10^{-3}	0.9943
	70	5	0.034	-	-	-	-	3.03×10^{-2}	1.02×10^{-3}	0.9904
	50	10	0.027	-	-	-	-	2.46×10^{-2}	0.66×10^{-3}	0.9934
	60	10	0.032	-	-	-	-	2.59×10^{-2}	0.75×10^{-3}	0.9929
	70	10	0.033	-	-	-	-	3.39×10^{-2}	1.29×10^{-3}	0.9889
**Page**	50	0	-	1.353	0.006	-	-	0.61×10^{-2}	0.42×10^{-4}	0.9995
	60	0	-	1.370	0.007	-	-	0.74×10^{-2}	0.63×10^{-4}	0.9992
	70	0	-	1.612	0.003	-	-	0.42×10^{-2}	0.21×10^{-4}	0.9998
	50	5	-	1.162	0.013	-	-	0.69×10^{-2}	0.56×10^{-4}	0.9994
	60	5	-	1.415	0.007	-	-	0.88×10^{-2}	0.93×10^{-4}	0.9991
	70	5	-	1.632	0.003	-	-	0.48×10^{-2}	0.28×10^{-4}	0.9998
	50	10	-	1.353	0.007	-	-	0.46×10^{-2}	0.26×10^{-4}	0.9998
	60	10	-	1.458	0.006	-	-	1.19×10^{-2}	1.77×10^{-4}	0.9985
	70	10	-	1.667	0.003	-	-	0.69×10^{-2}	0.61×10^{-4}	0.9995
**Henderson and Pabis**	50	0	0.025	-	1.019	-	-	2.19×10^{-2}	0.55×10^{-3}	0.9932
	60	0	0.027	-	1.015	-	-	2.14×10^{-2}	0.53×10^{-3}	0.9936
	70	0	0.031	-	1.015	-	-	2.99×10^{-2}	1.07×10^{-3}	0.9894
	50	5	0.025	-	1.010	-	-	1.29×10^{-2}	0.19×10^{-3}	0.9978
	60	5	0.032	-	1.009	-	-	2.13×10^{-2}	0.54×10^{-3}	0.9944
	70	5	0.034	-	1.012	-	-	3.00×10^{-2}	1.13×10^{-3}	0.9905
	50	10	0.027	-	1.015	-	-	2.41×10^{-2}	0.71×10^{-3}	0.9936
	60	10	0.032	-	1.010	-	-	2.57×10^{-2}	0.83×10^{-3}	0.9930
	70	10	0.033	-	1.013	-	-	3.36×10^{-2}	1.45×10^{-3}	0.9891
**Logarithmic**	50	0	0.025	-	1.022	-	-0.004	2.16×10^{-2}	0.58×10^{-3}	0.9933
	60	0	0.027	-	1.019	-	-0.004	2.11×10^{-2}	0.56×10^{-3}	0.9938
	70	0	0.031	-	1.023	-	-0.008	2.91×10^{-2}	1.13×10^{-3}	0.9900
	50	5	0.025	-	1.010	-	-0.001	1.28×10^{-2}	0.21×10^{-3}	0.9978
	60	5	0.032	-	1.012	-	-0.003	2.12×10^{-2}	0.60×10^{-3}	0.9945
	70	5	0.033	-	1.020	-	-0.009	2.92×10^{-2}	1.22×10^{-3}	0.9911
	50	10	0.026	-	1.023	-	-0.009	2.32×10^{-2}	0.74×10^{-3}	0.9941
	60	10	0.032	-	1.014	-	-0.004	2.56×10^{-2}	0.93×10^{-3}	0.9931
	70	10	0.032	-	1.024	-	-0.012	3.24×10^{-2}	1.58×10^{-3}	0.9899
**Wang and Singh**	50	0	-	-	-0.81×10^{-2}	0.14×10^{-4}	-	0.1752	0.0351	0.5621
	60	0	-	-	-0.87×10^{-2}	0.16×10^{-4}	-	0.1774	0.0363	0.5612
	70	0	-	-	-1.09×10^{-2}	0.25×10^{-4}	-	0.1659	0.0330	0.6739
	50	5	-	-	-0.91×10^{-2}	0.18×10^{-4}	-	0.1575	0.0289	0.6706
	60	5	-	-	-1.08×10^{-2}	0.25×10^{-4}	-	0.1698	0.0346	0.6469
	70	5	-	-	-1.30×10^{-2}	0.37×10^{-4}	-	0.1491	0.0278	0.7667
	50	10	-	-	-1.14×10^{-2}	0.29×10^{-4}	-	0.1316	0.0212	0.8104

	60	10	-	-	-1.27×10^{-2}	0.36×10^{-4}	-	0.1436	0.0258	0.7810
	70	10	-	-	-1.42×10^{-2}	0.44×10^{-4}	-	0.1303	0.0218	0.8360
**Midilli et al.**	50	0	0.006	1.341	0.997	0.143×10^{-4}	-	0.73×10^{-2}	0.71×10^{-4}	0.9992
	60	0	0.007	1.346	0.998	0.113×10^{-4}	-	0.72×10^{-2}	0.72×10^{-4}	0.9993
	70	0	0.003	1.597	0.999	0.101×10^{-4}	-	0.41×10^{-2}	0.25×10^{-4}	0.9998
	50	5	0.014	1.152	0.998	0.150×10^{-4}	-	0.73×10^{-2}	0.74×10^{-4}	0.9993
	60	5	0.008	1.380	0.998	0.234×10^{-4}	-	0.90×10^{-2}	1.20×10^{-4}	0.9990
	70	5	0.004	1.582	0.997	0.190×10^{-4}	-	0.53×10^{-2}	0.47×10^{-4}	0.9997
	50	10	0.007	1.335	0.997	0.219×10^{-4}	-	0.64×10^{-2}	0.64×10^{-4}	0.9996
	60	10	0.007	1.416	0.997	0.350×10^{-4}	-	1.13×10^{-2}	2.12×10^{-4}	0.9986
	70	10	0.003	1.673	1.000	0.216×10^{-4}	-	0.63×10^{-2}	0.71×10^{-4}	0.9996
**Diffusion Approach**	50	0	0.025	-	0.698	1.000	-	2.24×10^{-2}	6.19×10^{-4}	0.9928
	60	0	0.027	-	0.705	1.000	-	2.17×10^{-2}	5.91×10^{-4}	0.9934
	70	0	0.031	-	0.595	1.000	-	3.02×10^{-2}	12.18×10^{-4}	0.9892
	50	5	0.025	-	0.648	0.999	-	1.31×10^{-2}	2.19×10^{-4}	0.9977
	60	5	0.032	-	0.688	1.000	-	2.15×10^{-2}	6.15×10^{-4}	0.9943
	70	5	0.034	-	0.609	1.000	-	3.03×10^{-2}	13.09×10^{-4}	0.9904
	50	10	0.027	-	0.639	1.003	-	2.46×10^{-2}	8.31×10^{-4}	0.9934
	60	10	0.032	-	0.633	1.008	-	2.59×10^{-2}	9.60×10^{-4}	0.9929
	70	10	0.033	-	0.601	1.000	-	3.39×10^{-2}	17.19×10^{-3}	0.9889

Note: T - temperature (°C); EA - egg white albumen concentration (%); *a*, *b*, *c*, *n*, *k* - specific coefficients for each model; R^2 - coefficient of determination; RMSE - root mean square errors, χ^2 - chi-square

Table S2. Mathematical models applied to the experimental data for IR drying

Mathematical model	T, °C	EA, %	k	n	a	b	c	RMSE	χ^2	R^2
**Lewis/ Newtown**	50	0	0.021	-	-	-	-	3.21×10^{-2}	1.10×10^{-3}	0.9871
	60	0	0.022	-	-	-	-	2.86×10^{-2}	0.88×10^{-3}	0.9900
	70	0	0.024	-	-	-	-	4.19×10^{-2}	1.91×10^{-3}	0.9811
	50	5	0.012	-	-	-	-	4.70×10^{-2}	2.39×10^{-3}	0.9788
	60	5	0.019	-	-	-	-	3.20×10^{-2}	1.11×10^{-3}	0.9893
	70	5	0.023	-	-	-	-	4.46×10^{-2}	2.21×10^{-3}	0.9812
	50	10	0.014	-	-	-	-	5.12×10^{-2}	2.86×10^{-3}	0.9755
	60	10	0.021	-	-	-	-	3.92×10^{-2}	1.71×10^{-3}	0.9855
	70	10	0.022	-	-	-	-	5.46×10^{-2}	3.35×10^{-3}	0.9745
**Page**	50	0	-	1.427	0.004	-	-	0.92×10^{-2}	0.98×10^{-4}	0.9989
	60	0	-	1.386	0.005	-	-	0.55×10^{-2}	0.35×10^{-4}	0.9996
	70	0	-	1.557	0.003	-	-	1.27×10^{-2}	1.94×10^{-4}	0.9983
	50	5	-	1.404	0.002	-	-	1.45×10^{-2}	2.49×10^{-4}	0.9980
	60	5	-	1.328	0.005	-	-	0.96×10^{-2}	1.11×10^{-4}	0.9990
	70	5	-	1.554	0.003	-	-	0.82×10^{-2}	0.85×10^{-4}	0.9994
	50	10	-	1.478	0.002	-	-	1.51×10^{-2}	2.74×10^{-4}	0.9979
	60	10	-	1.412	0.004	-	-	0.96×10^{-2}	1.16×10^{-4}	0.9991
	70	10	-	1.620	0.002	-	-	0.88×10^{-2}	1.00×10^{-4}	0.9993
	50	0	0.021	-	1.030	-	-	3.10×10^{-2}	1.11×10^{-3}	0.9879

Henderson and Pabis	60	0	0.022	-	1.026	-	-	2.76×10^{-2}	0.89×10^{-3}	0.9907
	70	0	0.024	-	1.031	-	-	4.09×10^{-2}	2.00×10^{-3}	0.9820
	50	5	0.013	-	1.057	-	-	4.34×10^{-2}	2.22×10^{-3}	0.9820
	60	5	0.019	-	1.031	-	-	3.05×10^{-2}	1.12×10^{-3}	0.9902
	70	5	0.024	-	1.032	-	-	4.33×10^{-2}	2.35×10^{-3}	0.9822
	50	10	0.015	-	1.056	-	-	4.79×10^{-2}	2.75×10^{-3}	0.9786
	60	10	0.021	-	1.031	-	-	3.78×10^{-2}	1.79×10^{-3}	0.9865
	70	10	0.022	-	1.040	-	-	5.27×10^{-2}	3.58×10^{-3}	0.9762
Logarithmic	50	0	0.021	-	1.038	-	-0.010	3.01×10^{-2}	1.13×10^{-3}	0.9886
	60	0	0.022	-	1.034	-	-0.010	2.66×10^{-2}	0.90×10^{-3}	0.9914
	70	0	0.023	-	1.046	-	-0.017	3.88×10^{-2}	2.01×10^{-3}	0.9838
	50	5	0.011	-	1.093	-	-0.050	3.53×10^{-2}	1.62×10^{-3}	0.9881
	60	5	0.018	-	1.046	-	-0.019	2.78×10^{-2}	1.03×10^{-3}	0.9919
	70	5	0.022	-	1.053	-	-0.025	3.99×10^{-2}	2.28×10^{-3}	0.9849
	50	10	0.013	-	1.091	-	-0.046	4.09×10^{-2}	2.23×10^{-3}	0.9844
	60	10	0.019	-	1.055	-	-0.030	3.32×10^{-2}	1.58×10^{-3}	0.9896
	70	10	0.020	-	1.076	-	-0.043	4.65×10^{-2}	3.25×10^{-3}	0.9815
Wang and Singh	50	0	-	-	-0.84×10^{-2}	0.15×10^{-4}	-	0.1456	0.0245	0.7333
	60	0	-	-	-0.90×10^{-2}	0.18×10^{-4}	-	0.1407	0.0231	0.7581
	70	0	-	-	-1.05×10^{-2}	0.24×10^{-4}	-	0.1287	0.0199	0.8213
	50	5	-	-	-0.80×10^{-2}	0.15×10^{-4}	-	0.0442	0.0023	0.9812
	60	5	-	-	-0.98×10^{-2}	0.21×10^{-4}	-	0.0916	0.0101	0.9119
	70	5	-	-	-1.21×10^{-2}	0.33×10^{-4}	-	0.0948	0.0112	0.9149
	50	10	-	-	-0.90×10^{-2}	0.19×10^{-4}	-	0.0535	0.0034	0.9733
	60	10	-	-	-1.16×10^{-2}	0.31×10^{-4}	-	0.0749	0.0070	0.9472
	70	10	-	-	-1.28×10^{-2}	0.37×10^{-4}	-	0.0658	0.0056	0.9630
Midilli et al.	50	0	0.004	1.433	0.997	0.083×10^{-4}	-	0.93×10^{-2}	1.18×10^{-4}	0.9989
	60	0	0.005	1.388	0.999	0.077×10^{-4}	-	0.56×10^{-2}	0.43×10^{-4}	0.9996
	70	0	0.003	1.561	0.998	0.006×10^{-4}	-	1.27×10^{-2}	2.43×10^{-4}	0.9983
	50	5	0.002	1.429	0.986	0.086×10^{-4}	-	1.33×10^{-2}	2.56×10^{-4}	0.9983
	60	5	0.005	1.329	0.996	0.077×10^{-4}	-	1.00×10^{-2}	1.49×10^{-4}	0.9990
	70	5	0.003	1.544	0.995	0.063×10^{-4}	-	1.04×10^{-2}	1.82×10^{-4}	0.9990
	50	10	0.002	1.500	0.988	0.045×10^{-4}	-	1.44×10^{-2}	3.11×10^{-4}	0.9981
	60	10	0.004	1.406	0.994	0.018×10^{-4}	-	1.15×10^{-2}	2.22×10^{-4}	0.9987
	70	10	0.002	1.613	0.995	0.008×10^{-4}	-	1.08×10^{-2}	2.11×10^{-4}	0.9990
Diffusion Approach	50	0	0.021	-	0.644	1.000	-	3.21×10^{-2}	1.29×10^{-3}	0.9871
	60	0	0.022	-	0.727	1.000	-	2.86×10^{-2}	1.04×10^{-3}	0.9900
	70	0	0.024	-	0.620	1.000	-	4.19×10^{-2}	2.34×10^{-3}	0.9811
	50	5	0.012	-	0.516	1.000	-	4.70×10^{-2}	2.87×10^{-3}	0.9788
	60	5	0.019	-	0.538	0.999	-	3.20×10^{-2}	1.36×10^{-3}	0.9893
	70	5	0.023	-	0.530	0.999	-	4.46×10^{-2}	2.84×10^{-3}	0.9812
	50	10	0.014	-	0.507	1.000	-	5.12×10^{-2}	3.49×10^{-3}	0.9755
	60	10	0.020	-	0.529	1.008	-	3.92×10^{-2}	2.20×10^{-3}	0.9855
	70	10	0.022	-	0.524	1.007	-	5.46×10^{-2}	4.47×10^{-3}	0.9745

Note: T - temperature (°C); EA - egg white albumen concentration (%); *a*, *b*, *c*, *n*, *k* - specific coefficients for each model; R^2 - coefficient of determination; RMSE - root mean square errors, χ^2 - chi-square

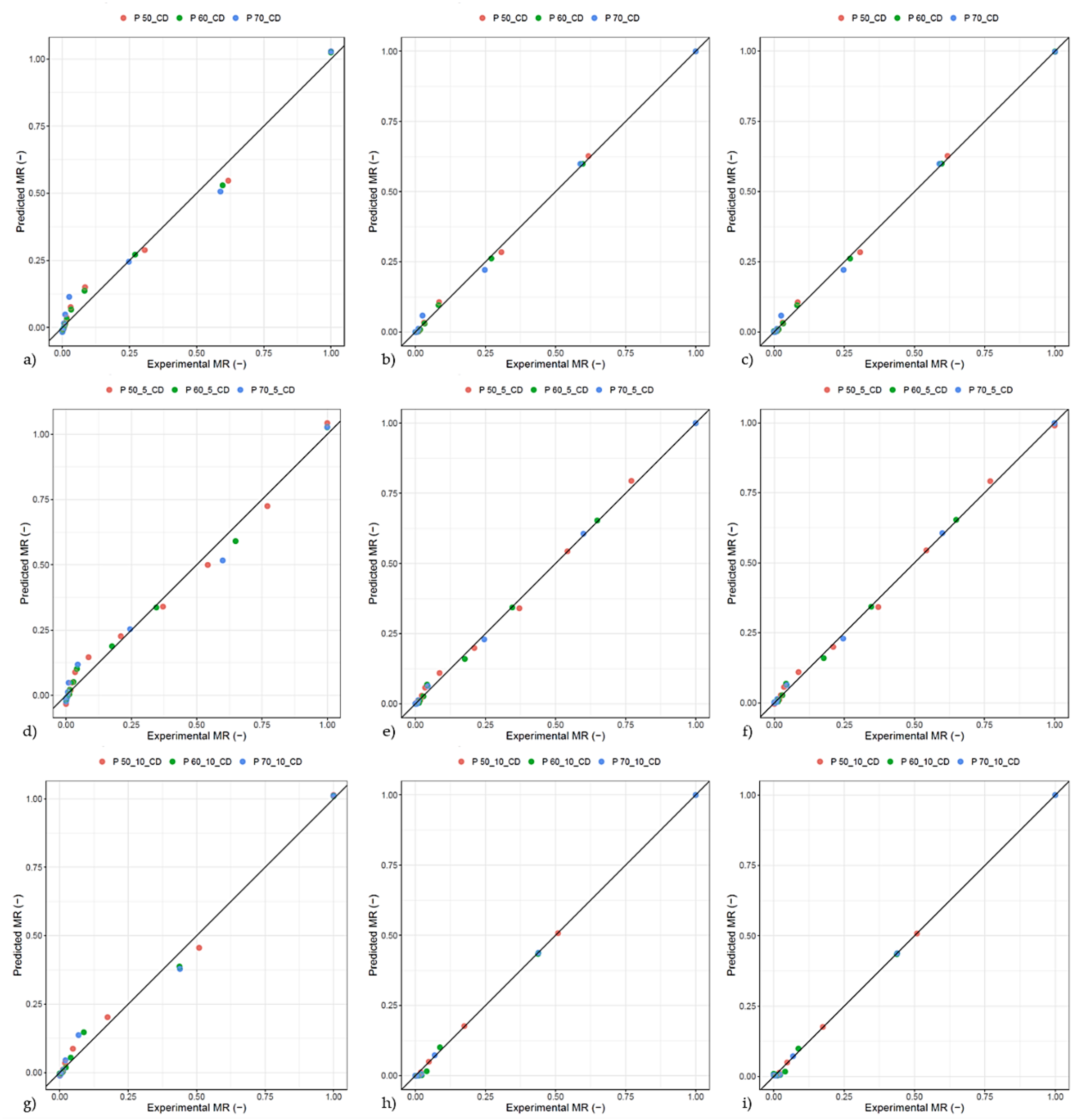

Figure S1. Predicted versus experimental (MR) values for CD blackthorn purée with 0%, 5% and 10% EA, fitted using the Logarithmic model (a, d, g), the Page model (b, e, h) and the Midilli model (c, f, i)

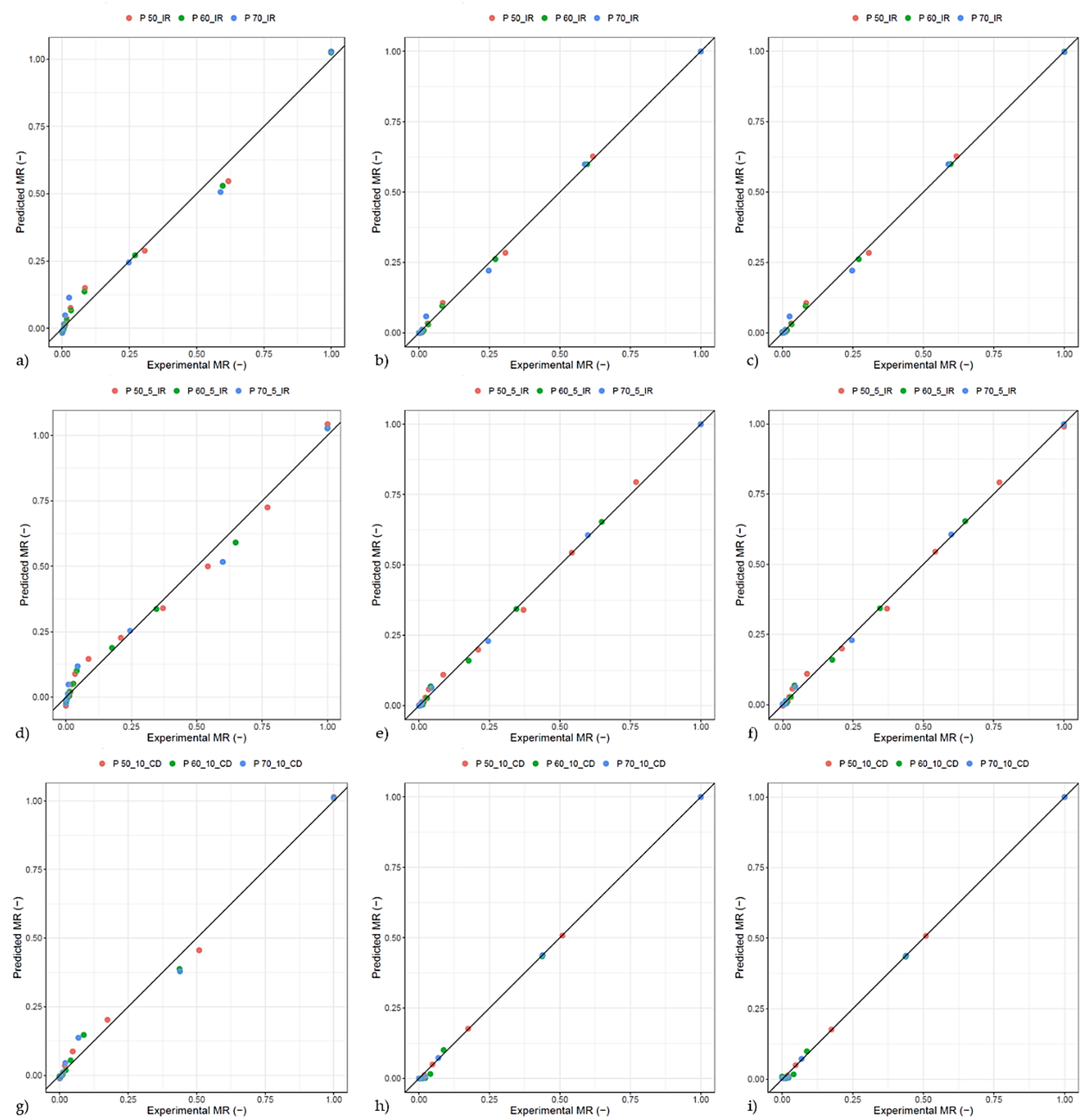

Figure S2. Predicted versus experimental (MR) values for IR blackthorn purée with 0%, 5% and 10% EA, fitted using the Logarithmic model (a, d, g), the Page model (b, e, h) and the Midilli model (c, f, i)

Table S3. Pearson correlation for the CD-dried blackthorn powders

	**T, ℃**	**EA, %**	**L*_CD**	**a*_CD**	**b*_CD**	**ΔE_CD**	**BI_CD**	**DT**	**DW**	**BD**	**TD**	**CI**	**HR**	**TAC**	**TPC**	**TFC**	**DPPH**
**T, ℃**	1.000	0.000	0.140	-0.139	0.287	0.341	-0.088	**-0.596**	**0.694**	**0.430**	**0.695**	0.350	0.363	**-0.402**	0.366	-0.185	0.087
**EA, %**	0.000	1.000	**0.621**	**0.730**	**-0.558**	**0.533**	**0.739**	**-0.775**	**0.462**	**-0.385**	-0.161	0.295	0.266	**-0.683**	**-0.770**	**-0.739**	**-0.736**
**L*_CD**	0.140	**0.621**	1.000	**0.825**	**-0.769**	-0.040	**0.828**	**-0.616**	**0.550**	**-0.411**	-0.026	**0.530**	**0.529**	**-0.554**	**-0.717**	**-0.678**	**-0.693**
**a*_CD**	-0.139	**0.730**	**0.825**	1.000	**-0.940**	-0.104	**0.986**	**-0.496**	**0.400**	**-0.477**	-0.168	**0.422**	**0.405**	**-0.654**	**-0.866**	**-0.747**	**-0.733**
**b*_CD**	0.287	**-0.558**	**-0.769**	**-0.940**	1.000	**0.384**	**-0.917**	0.312	-0.245	**0.427**	0.142	**-0.390**	-0.373	**0.460**	**0.797**	**0.580**	**0.552**
**ΔE_CD**	0.341	**0.533**	-0.040	-0.104	**0.384**	1.000	-0.078	**-0.549**	0.306	0.005	-0.001	-0.016	-0.024	-0.323	-0.075	-0.280	-0.309
**BI_CD**	-0.088	**0.739**	**0.828**	**0.986**	**-0.917**	-0.078	1.000	**-0.522**	**0.410**	**-0.482**	-0.184	**0.400**	**0.387**	**-0.728**	**-0.861**	**-0.785**	**-0.758**
**DT**	**-0.596**	**-0.775**	**-0.616**	**-0.496**	0.312	**-0.549**	**-0.522**	1.000	**-0.837**	0.055	-0.325	**-0.505**	**-0.485**	**0.692**	0.342	**0.615**	**0.444**
**DW**	**0.694**	**0.462**	**0.550**	**0.400**	-0.245	0.306	**0.410**	**-0.837**	1.000	-0.110	**0.419**	**0.719**	**0.709**	**-0.515**	-0.068	-0.329	-0.183
**BD**	**0.430**	**-0.385**	**-0.411**	**-0.477**	**0.427**	0.005	**-0.482**	0.055	-0.110	1.000	**0.751**	-0.362	-0.359	0.182	**0.466**	0.180	0.356
**TD**	**0.695**	-0.161	-0.026	-0.168	0.142	-0.001	-0.184	-0.325	**0.419**	**0.751**	1.000	0.342	0.342	-0.079	0.354	0.028	0.319
**CI**	0.350	0.295	**0.530**	**0.422**	**-0.390**	-0.016	**0.400**	**-0.505**	**0.719**	-0.362	0.342	1.000	**0.997**	-0.340	-0.154	-0.194	-0.039
**HR**	0.363	0.266	**0.529**	**0.405**	-0.373	-0.024	**0.387**	-0.485	**0.709**	-0.359	0.342	**0.997**	1.000	-0.338	-0.142	-0.196	-0.039
**TAC**	**-0.402**	**-0.683**	**-0.554**	**-0.654**	**0.460**	-0.323	**-0.728**	**0.692**	**-0.515**	0.182	-0.079	-0.340	-0.338	1.000	**0.564**	**0.882**	**0.669**
**TPC**	0.366	**-0.770**	**-0.717**	**-0.866**	**0.797**	-0.075	**-0.861**	0.342	-0.068	**0.466**	0.354	-0.154	-0.142	**0.564**	1.000	**0.773**	**0.875**
**TFC**	-0.185	**-0.739**	**-0.678**	**-0.747**	**0.580**	-0.280	**-0.785**	**0.615**	-0.329	0.180	0.028	-0.194	-0.196	**0.882**	**0.773**	1.000	**0.832**
**DPPH**	0.087	**-0.736**	**-0.693**	**-0.733**	**0.552**	-0.309	**-0.758**	**0.444**	-0.183	0.356	0.319	-0.039	-0.039	**0.669**	**0.875**	**0.832**	1.000

Note: T – temperature, ℃; EA – egg white albumen; BI - Brown Index; DT – drying time, min; DW - dry weight, %; BD -bulk density, $g \cdot mL^{-1}$; TD – tapped density, $g \cdot mL^{-1}$; CI – Carr`s Index, %; HR - Hausner Ratio; TAC – Total Anthocyanins Content, mg cyanidin-3-glicoside·100 g^{-1} DW, TPC – Total Polyphenolic Content, mg gallic acid equivalent (GAE)·g^{-1} DW, Antioxidant activity as DPPH, µmol Trolox·g^{-1} DW

Table S4. Pearson correlation for the IR-dried blackthorn powders

	**T, ℃**	**EA, %**	**L*_IR**	**a*_IR**	**b*_IR**	**ΔE_IR**	**BI_IR**	**DT**	**DW**	**BD**	**TD**	**CI**	**HR**	**TAC**	**TPC**	**TFC**	**DPPH**
**T, ℃**	1,000	0,000	-0,071	**-0,675**	0,335	0,258	**-0,512**	**-0,661**	**0,400**	**0,692**	**0,761**	-0,087	-0,073	0,328	-0,160	-0,016	-0,140
**EA, %**	0,000	1,000	**0,822**	-0,275	-0,370	0,120	**-0,521**	**-0,735**	**0,806**	0,181	0,080	-0,304	-0,323	**-0,726**	**-0,524**	**-0,707**	**-0,812**
**L*_IR**	-0,071	**0,822**	1,000	**-0,489**	-0,359	-0,017	**-0,683**	**-0,536**	**0,583**	0,296	0,114	**-0,523**	**-0,530**	**-0,601**	**-0,657**	**-0,822**	**-0,851**
**a*_IR**	**-0,675**	-0,275	**-0,489**	1,000	-0,003	0,013	**0,897**	**0,638**	**-0,412**	**-0,799**	**-0,660**	**0,602**	**0,586**	-0,051	**0,676**	**0,607**	**0,644**
**b*_IR**	0,335	-0,370	-0,359	-0,003	1,000	**0,856**	-0,039	0,108	0,034	**0,458**	**0,588**	0,182	0,188	0,201	0,145	0,284	0,253
**ΔE_IR**	0,258	0,120	-0,017	0,013	**0,856**	1,000	-0,162	-0,216	**0,438**	**0,479**	**0,581**	0,110	0,103	-0,168	-0,042	0,035	-0,091
**BI_IR**	**-0,512**	**-0,521**	**-0,683**	**0,897**	-0,039	-0,162	1,000	**0,693**	**-0,595**	**-0,766**	**-0,617**	**0,605**	**0,601**	0,280	**0,746**	**0,793**	**0,803**
**DT**	**-0,661**	**-0,735**	**-0,536**	**0,638**	0,108	-0,216	**0,693**	1,000	**-0,864**	**-0,578**	**-0,537**	0,310	0,316	0,301	**0,492**	**0,509**	**0,697**
**DW**	**0,400**	**0,806**	**0,583**	**-0,412**	0,034	**0,438**	**-0,595**	**-0,864**	1,000	**0,481**	**0,426**	-0,295	-0,316	**-0,514**	**-0,451**	**-0,512**	**-0,695**
**BD**	**0,692**	0,181	0,296	**-0,799**	**0,458**	**0,479**	**-0,766**	**-0,578**	**0,481**	1,000	**0,927**	**-0,499**	**-0,489**	0,008	**-0,509**	**-0,423**	**-0,501**
**TD**	**0,761**	0,080	0,114	**-0,660**	**0,588**	**0,581**	**-0,617**	**-0,537**	**0,426**	**0,927**	1,000	-0,139	-0,128	0,072	-0,290	-0,239	-0,291
**CI**	-0,087	-0,304	**-0,523**	**0,602**	0,182	0,110	**0,605**	0,310	-0,295	**-0,499**	-0,139	1,000	**0,998**	0,150	**0,669**	**0,578**	**0,653**
**HR**	-0,073	-0,323	**-0,530**	**0,586**	0,188	0,103	**0,601**	0,316	-0,316	**-0,489**	-0,128	**0,998**	1,000	0,169	**0,670**	**0,577**	**0,661**
**TAC**	0,328	**-0,726**	**-0,601**	-0,051	0,201	-0,168	0,280	0,301	**-0,514**	0,008	0,072	0,150	0,169	1,000	0,103	**0,601**	**0,459**
**TPC**	-0,160	**-0,524**	**-0,657**	**0,676**	0,145	-0,042	**0,746**	**0,492**	**-0,451**	**-0,509**	-0,290	**0,669**	**0,670**	0,103	1,000	**0,746**	**0,900**
**TFC**	-0,016	**-0,707**	**-0,822**	**0,607**	0,284	0,035	**0,793**	**0,509**	**-0,512**	**-0,423**	-0,239	**0,578**	**0,577**	**0,601**	**0,746**	1,000	**0,876**
**DPPH**	-0,140	**-0,812**	**-0,851**	**0,644**	0,253	-0,091	**0,803**	**0,697**	**-0,695**	**-0,501**	-0,291	**0,653**	**0,661**	**0,459**	**0,900**	**0,876**	1,000

Note: T – temperature, ℃; EA – egg white albumen; BI - Brown Index; DT – drying time, min; DW - dry weight, %; BD -bulk density, g·mL^{-1}; TD – tapped density, g·mL^{-1}; CI – Carr`s Index, %; HR - Hausner Ratio; TAC – Total Anthocyanins Content, mg cyanidin-3-glicoside·100 g^{-1} DW, TPC – Total Polyphenolic Content, mg gallic acid equivalent (GAE)·g^{-1} DW, Antioxidant activity as DPPH, μmol Trolox·g^{-1} DW

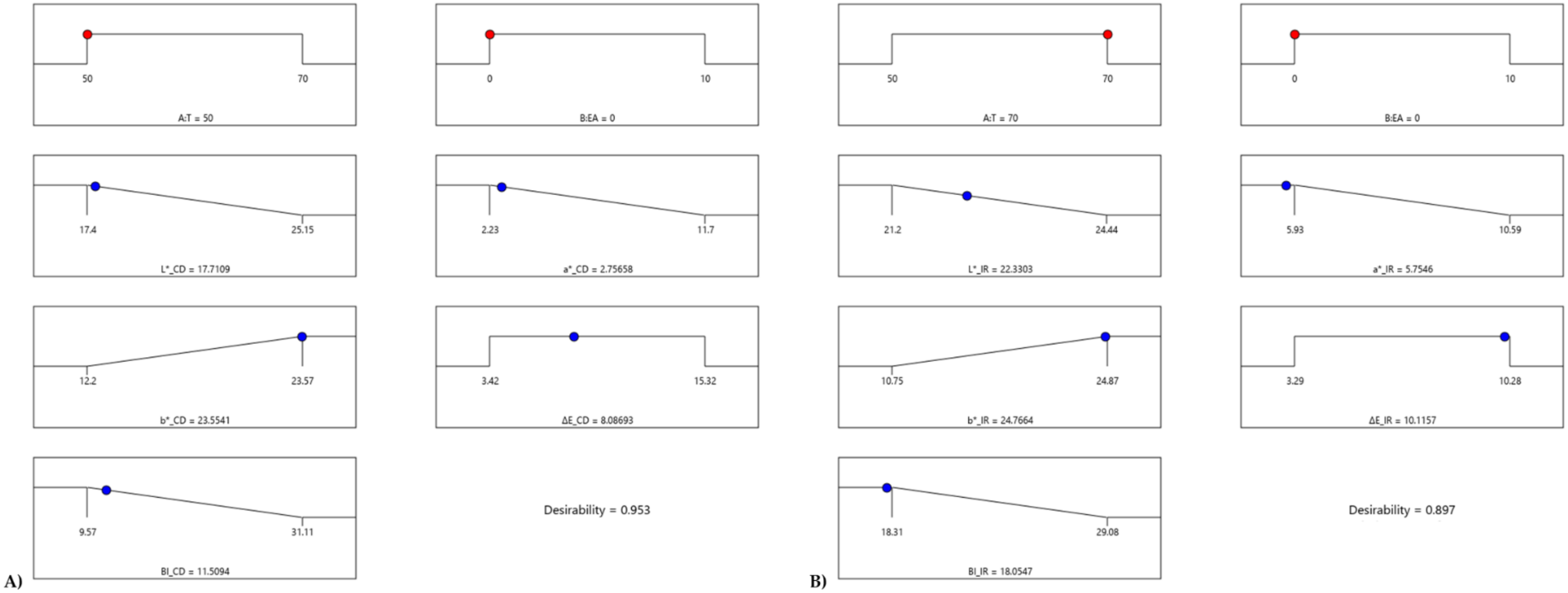

Figure S3. The constraints applied to the dependent variables (blue dots) and the independent variables associated with them (red dots) for CD-dried (A) and IR-dried (B) blackthorn powders

Table S5. Solutions identified for the optimization of CD drying conditions of blackthorn purée based on color parameters.

Number	T	EA	L*_CD	a*_CD	b*_CD	ΔE_CD	BI_CD	Desirability	
**1**	**50.000**	**0.000**	**17.711**	**2.757**	**23.554**	**8.087**	**11.509**	**0.953**	**Selected**
**2**	50.000	0.091	17.873	2.936	23.298	7.971	11.894	0.933	
**3**	51.686	0.000	18.106	3.356	22.470	7.161	13.010	0.883	
**4**	70.000	0.000	18.541	3.685	21.752	5.802	14.706	0.824	
**5**	70.000	0.000	18.541	3.685	21.752	5.802	14.706	0.824	
**6**	53.500	0.000	18.464	3.894	21.495	6.316	14.371	0.820	
**7**	68.500	0.000	18.771	4.084	21.050	5.315	15.573	0.781	
**8**	70.000	10.000	21.871	6.893	21.004	15.094	21.693	0.519	
**9**	69.087	10.000	22.147	7.319	20.150	14.326	22.659	0.471	
**10**	50.000	10.000	23.901	9.775	13.816	7.349	27.636	0.166	

Table S6. Solutions identified for the optimization of IR drying conditions of blackthorn purée based on color parameters.

Number	T	EA	L*_IR	a*_IR	b*_IR	ΔE_IR	BI_IR	Desirability	
**1**	**70.000**	**0.000**	**22.330**	**5.755**	**24.766**	**10.116**	**18.055**	**0.897**	**Selected**
**2**	69.740	0.000	22.336	5.787	24.532	9.921	18.134	0.892	
**3**	70.000	0.102	22.376	5.853	24.560	10.013	18.286	0.889	
**4**	69.430	0.000	22.343	5.824	24.258	9.693	18.226	0.887	
**5**	70.000	0.174	22.407	5.922	24.415	9.941	18.449	0.880	
**6**	68.500	0.000	22.358	5.930	23.469	9.037	18.491	0.869	
**7**	50.000	0.000	21.264	6.068	18.241	4.772	19.945	0.809	
**8**	50.594	0.000	21.340	6.122	18.100	4.650	20.011	0.796	
**9**	53.500	0.000	21.675	6.329	17.702	4.297	20.227	0.750	
**10**	54.639	0.000	21.788	6.384	17.680	4.272	20.262	0.738	
**11**	70.000	10.000	22.737	7.648	12.228	7.349	23.095	0.373	
**12**	50.000	10.000	23.670	9.321	14.873	9.026	26.525	0.259	
**13**	50.079	10.000	23.673	9.323	14.817	8.981	26.528	0.257	

Table S7. Experimental confirmation (n = 3) of optimal independent variable for CD and IR drying methods.

Analysis	Predicted Mean	95% CI	Data Mean
**L*_CD**	17.71	16.26 - 19.17	17.50 ± 3.52
**a*_CD**	2.76	0.37 - 5.14	2.26 ± 0.15
**b*_CD**	23.55	23.04 - 24.07	23.62 ± 4.63
**ΔE_CD**	8.09	7.05 - 9.12	7.99 ± 0.84
**BI_CD**	11.51	5.18 - 17.84	9.62 ± 0.25
**L*_IR**	22.33	21.31 - 23.35	21.99 ± 1.56
**a*_IR**	5.76	4.12 - 7.39	5.44 ± 0.46
**b*_IR**	24.77	24.13 - 25.41	24.86 ± 3.45
**ΔE_IR**	10.12	9.16 - 11.67	9.96 ± 0.63
**BI_IR**	18.06	14.68 - 21.44	17.25 ± 0.96

Note: CI – confidence interval; n – number of measurements

Table S8. Eigenvalue analysis of the correlation matrix calculated for the dependent variables

	PC1	PC2	PC3	PC4	PC5	PC6	PC7	PC8	PC9	PC10	PC11	PC12	PC13	PC14
**Eigenvalue**	7.5400	3.7822	2.8571	1.5752	0.7258	0.4733	0.3244	0.2854	0.2447	0.0766	0.0440	0.0270	0.0267	0.0123
**Proportion, %**	41.9	21.0	15.9	8.8	4.0	2.6	1.8	1.6	1.4	0.4	0.4	0.3	0.2	0.1
**Cumulative, %**	41.9	62.9	78.8	87.5	91.6	94.2	96.0	97.6	98.9	99.4	99.6	99.8	99.9	100

Table S9. Eigenvectors corresponding to principal components with eigenvalues greater than 1

Variable	PC1	PC2	PC3	PC4
Temperature, ℃ (T)	0.085	-0.463	-0.025	0.111
Egg albumin, % (EA)	0.322	0.045	-0.017	-0.299
Moisture effective diffusivity, $m^2 \cdot s^{-1}$ (D_{eff})	0.218	-0.380	0.031	-0.155
Lightness (L*)	0.295	0.168	0.145	0.099
Redness/blueness (a*)	0.263	0.089	0.161	0.306
Yellowness/Greenness (b*)	-0.283	-0.246	0.101	-0.215
Total difference color (ΔE)	0.052	-0.260	0.138	-0.620
Browning Index (BI)	0.303	0.231	0.065	0.083
Drying time, min (DT)	-0.303	0.249	-0.025	0.109
Dry weight, % (DW)	0.288	-0.235	0.087	0.049
Bulk density, $g \cdot mL^{-1}$ (BD)	0.036	-0.309	-0.403	0.269
Tapped density, $g \cdot mL^{-1}$ (TD)	0.078	-0.386	-0.177	0.384
Carr`s index, % (CI)	0.086	-0.087	0.517	0.176
Haussner Ratio, (HR)	0.068	-0.088	0.537	0.183
Total anthocyanins content, mg C3G·100 g^{-1} DW (TAC)	-0.245	-0.080	-0.085	0.127
Total polyphenolic content, mg GAE·g^{-1} DW (TPC)	-0.250	-0.164	0.274	-0.001
Total flavonoid content, mg EQ·g^{-1} DW (TFC)	-0.291	-0.057	0.218	0.101
Antioxidant activity, μmol Trolox·g^{-1} DW (DPPH)	-0.331	-0.087	0.173	0.091

Table S10. Chromatographic analysis of extracts obtained from powders dried at 60 °C using CD and IR, with 0%, 5%, and 10% EA concentrations.

Compound	60℃ without EA		60℃ with 5% EA		60℃ with 10% EA	
	CD	IR	CD	IR	CD	IR
**Phenolic acids**						
**Gallic acid**	7.73 ± 0.54 ^{B}	n.d.	11.22 ± 0.96^{A}	n.d.	n.d.	n.d.
**4-Hydroxybenzoic acid**	n.d.	n.d.	n.d.	0.54 ± 0.11	traces	traces
**Chlorogenic acid**	4.49 ± 0.02 $^{a, A}$	4.45 ± 0.21 $^{a, A}$	4.61 ± 0.34 ^{A}	traces	n.d.	5.32 ± 0.29 ^{A}
**Ellagic acid**	3.01 ± 0.05 ^{A}	n.d.	n.d.	n.d.	n.d.	3.38 ± 0.12 ^{A}
***p*-Coumaric acid**	n.d.	0.56 ± 0.01 ^{A}	n.d.	n.d.	n.d.	0.70 ± 0.09 ^{A}
**Cinnamic acid**	0.45 ± 0.03 $^{b, B}$	0.49 ± 0.02 $^{a, AB}$	0.45 ± 0.07 ^{B}	n.d.	0.64 ± 0.03 $^{a, A}$	0.48 ± 0.03 $^{b, B}$
**Vanillic acid**	traces	traces	traces	n.d.	traces	traces
**Sinapic acid**	traces	traces	n.d.	3.68 ± 0.22 ^{A}	1.76 ± 0.01 ^{B}	n.d.
**Syringic acid**	traces	n.d.	n.d.	n.d.	n.d.	n.d.
**Protocatechuic acid**	traces	traces	n.d.	15.47 ± 1.60	n.d.	n.d.
**Flavones**						
**Apigenin**	n.d.	n.d.	n.d.	0.77 ± 0.04 ^{A}	0.79 ± 0.11 ^{A}	n.d.
**Luteolin**	n.d.	n.d.	n.d.	0.94 ± 0.01 ^{A}	1.01 ± 0.06 ^{A}	n.d.
**Flavonols**						
**Quercetin 3-diglucoside**	1.33 ± 0.30 $^{a, A}$	1.63 ± 0.45 $^{a, A}$	n.d.	1.13 ± 0.03 ^{A}	1.14 ± 0.02 ^{A}	n.d.
**Quercetin 3-glucoside**	2.57 ± 0.43 ^{A}	n.d.	1.54 ± 0.11 $^{a, B}$	1.39 ± 0.10 $^{a, B}$	n.d.	1.62 ± 0.22 ^{B}
**Rutin trihydrate**	1.26 ± 0.12 ^{AB}	traces	n.d.	n.d.	0.92 ± 0.35 $^{b, B}$	1.64 ± 0.31 $^{a, A}$
**Kaempferol**	1.03 ± 0.01	n.d.	n.d.	n.d.	n.d.	n.d.
**Isorhamnetin**	0.49 ± 0.03 ^{A}	n.d.	n.d.	0.58 ± 0.07 ^{A}	n.d.	n.d.
**Flavanols**						
**Epigallocatechin**	247.86 ± 3.57 $^{a, A}$	228.49 ± 0.98 $^{b, B}$	250.52 ± 6.82 ^{A}	n.d.	208.74 ± 5.85 $^{b, C}$	220,63 ± 1.07 $^{a, C}$
**Catechin**	77.87 ± 0.98 $^{a, A}$	73.96 ± 0.34 $^{b, AB}$	72.40 ± 3.66 $^{b, BC}$	78.49 ± 4.33 $^{a, AB}$	70.39 ± 2.05 $^{a, C}$	72.29 ± 4.82 $^{a, C}$
**Epicatechin gallate**	2.51 ± 0.44 ^{B}	traces	n.d.	8.15 ± 0.97 ^{A}	2.96 ± 0.28 $^{b, B}$	3.98 ± 0.40 $^{a, B}$
**Flavanones**						
**Naringin**	2.57 ± 0.12 ^{A}	n.d.	n.d.	3.11 ± 0.17 ^{A}	0.63 ± 0.12 ^{B}	n.d.
**Hesperidin**	n.d.	n.d.	n.d.	3.27 ± 0.73 ^{A}	4.35 ± 0.72 ^{A}	n.d.
**Anthocyanins**						
**Peonidin 3-O-glucoside**	1.09 ± 0.01 ^{A}	n.d.	1.11 ± 0.01 ^{A}	n.d.	1.03 ± 0.17 $^{a, A}$	0.86 ± 0.01 $^{b, B}$
**Peonidin 3-O-rutinoside**	n.d.	n.d.	n.d.	traces	n.d.	n.d.
**Kuromanin (Cyanidin-3-O-glucoside)**	1.04 ± 0.06 $^{a, A}$	0.99 ± 0.05 $^{a, A}$	1.34 ± 0.07 $^{a, A}$	0.79 ± 0.22 $^{b, A}$	0.90 ± 0.14 $^{a, A}$	1.30 ± 0.47 $^{a, A}$
**Callistephin (Pelargonidin-3-glucoside)**	n.d.	0.94 ± 0.12 ^{A}	1.01 ± 0.04 $^{a, A}$	0.70 ± 0.08 $^{b, B}$	n.d.	0.73 ± 0.02 ^{B}
**Keracyanin (Cyanidin-3-O-galactoside)**	0.81 ± 0.02 ^{a}	0.75 ± 0.02 ^{b}	n.d.	n.d.	n.d.	traces
**Terpenoids**						
**Cafestol**	n.d.	n.d.	traces	n.d.	n.d.	n.d.
**Alkaloids**						
**Caffeine**	n.d.	0.82 ± 0.12	n.d.	n.d.	n.d.	n.d.

Note: **n.d.** – not detected; Different lowercase letters mean statistically significant differences between powders dried at the same temperature, but using different drying methods, while different uppercase letters mean statistically significant differences between powders for the same bioactive compound
